# Frequency discrimination and stimulus deviance in the inferior colliculus and cochlear nucleus

**DOI:** 10.3389/fncir.2012.00119

**Published:** 2013-01-14

**Authors:** Yaneri A. Ayala, David Pérez-González, Daniel Duque, Israel Nelken, Manuel S. Malmierca

**Affiliations:** ^1^Auditory Neurophysiology Laboratory, Institute of Neuroscience of Castilla y León, University of SalamancaSalamanca, Spain; ^2^Department of Neurobiology, Institute of Life Sciences, The Interdisciplinary Center for Neural Computation and the Edmond and Lily Safra Center for Brain Sciences, The Hebrew University of JerusalemJerusalem, Israel; ^3^Department of Cell Biology and Pathology, Faculty of Medicine, University of SalamancaSalamanca, Spain

**Keywords:** SSA, deviant sensitivity, change detection, mismatch negativity, non-lemniscal pathway, ROC analysis

## Abstract

Auditory neurons that exhibit stimulus-specific adaptation (SSA) decrease their response to common tones while retaining responsiveness to rare ones. We recorded single-unit responses from the inferior colliculus (IC) where SSA is known to occur and we explored for the first time SSA in the cochlear nucleus (CN) of rats. We assessed an important functional outcome of SSA, the extent to which frequency discriminability depends on sensory context. For this purpose, pure tones were presented in an oddball sequence as standard (high probability of occurrence) or deviant (low probability of occurrence) stimuli. To study frequency discriminability under different probability contexts, we varied the probability of occurrence and the frequency separation between tones. The neuronal sensitivity was estimated in terms of spike-count probability using signal detection theory. We reproduced the finding that many neurons in the IC exhibited SSA, but we did not observe significant SSA in our CN sample. We concluded that strong SSA is not a ubiquitous phenomenon in the CN. As predicted, frequency discriminability was enhanced in IC when stimuli were presented in an oddball context, and this enhancement was correlated with the degree of SSA shown by the neurons. In contrast, frequency discrimination by CN neurons was independent of stimulus context. Our results demonstrated that SSA is not widespread along the entire auditory pathway, and suggest that SSA increases frequency discriminability of single neurons beyond that expected from their tuning curves.

## Introduction

Auditory neurons displaying stimulus-specific adaptation (SSA) decrease their response to high probability stimuli (standards) while maintaining responsiveness to rare ones (deviants, Ulanovsky et al., 2003). SSA is correlated with behavioral habituation (Netser et al., 2011; Gutfreund, 2012) and it has been proposed to underlie sensory memory for stimulation history (Ulanovsky et al., 2004). Neurons showing SSA have been found in the mammalian auditory pathway from the inferior colliculus (IC) up to the cortex (Ulanovsky et al., 2003; Pérez-González et al., 2005; Anderson et al., 2009; Malmierca et al., 2009; von der Behrens et al., 2009; Yu et al., 2009; Antunes et al., 2010; Lumani and Zhang, 2010; Reches et al., 2010; Taaseh et al., 2011; Zhao et al., 2011; Ayala and Malmierca, 2012; Duque et al., 2012; Anderson and Malmierca, 2013) as well as in bird midbrain and forebrain (Reches and Gutfreund, 2008, 2009). Originally, SSA was suggested to emerge in the auditory cortex and to be transmitted downstream to subcortical nuclei through the corticofugal pathway (Nelken and Ulanovsky, 2007), as subcortical SSA is mostly confined to the non-lemniscal regions (Malmierca et al., 2009; Antunes et al., 2010; Duque et al., 2012), the main target of the corticofugal projections (Malmierca and Ryugo, 2011). However, it has been recently shown that cortical deactivation does not affect SSA neither in the non-lemniscal auditory thalamus (Antunes and Malmierca, 2011) nor in the IC (Anderson and Malmierca, 2013), while SSA in lemniscal regions is minimal (Malmierca et al., 2009; Antunes et al., 2010; Bäuerle et al., 2011). Thus, SSA may be computed independently in the non-lemniscal pathway and in primary auditory cortex. Thus far, the existence of SSA has not been explored in auditory nuclei below the IC, where the lemniscal and non-lemniscal divisions first emerge.

Frequency discrimination has been widely explored in psychoacoustics (Nelson and Kiester, 1978; Sinnott et al., 1985; Syka et al., 1996; Talwar and Gerstein, 1998, 1999; Shofner, 2000; Witte and Kipke, 2005; Walker et al., 2009), but few studies tested the detection of frequency deviants by single neurons (Ulanovsky et al., 2003; Malmierca et al., 2009; von der Behrens et al., 2009). SSA has already been shown to result in a change in frequency discrimination performance by single neurons (Ulanovsky et al., 2003; Malmierca et al., 2009) but this relationship has not been thoroughly explored.

The main goal of our study is to compare the relationships between frequency discrimination and SSA in two neuronal populations; one at the IC that it is already known to exhibit SSA and the other at a lower auditory structure, the cochlear nucleus (CN) where SSA has not been explored thus far. For this purpose we assessed whether the probabilistic context affects frequency discrimination as judged by signal detection theory (Green and Swets, 1966) based on distributions of spike counts, and to what extent changes in frequency discriminability reflect the degree of SSA in these two stations. We show that SSA and the enhancement in neurometric frequency discrimination in the IC are strongly correlated and that both depend on the frequency separation and deviant probability in similar ways. Our results also demonstrated that SSA and context-dependent neuronal sensitivity are not present in CN supporting the hypothesis that SSA first emerge in non-lemniscal IC.

## Materials and methods

### Surgical procedures

Experiments were performed on 71 adult female rats (*Rattus norvergicus*, Rj: Long–Evans) with body weights between 160 and 270 g. All experimental procedures were carried out at the University of Salamanca with the approval of, and using methods conforming to the standards of, the University of Salamanca Animal Care Committee. Anesthesia was induced (1.5 g/kg, i.p., 20% solution) and maintained (0.5 g/kg, i.p. given as needed) with urethane. Urethane was chosen as an anesthetic because of its effects on multiple aspects of neural activity, including inhibition and spontaneous firing, are known to be less than those of barbiturates and other anesthetic drugs (Hara and Harris, 2002). The respiration was maintained artificially (SAR-830/P Ventilator) monitoring the end-tidal CO_2_ level (CapStar-100). For this purpose, the trachea was cannulated and atropine sulfate (0.05 mg/kg, s.c.) was administered to reduce bronchial secretions. Body temperature was maintained at 38 ± 1°C by means of a heating blanket. Details of surgical procedures have been described previously (Hernández et al., 2005; Pérez-González et al., 2005; Malmierca et al., 2009; Antunes et al., 2010). The animal was placed inside a sound-attenuated room in a stereotaxic frame in which the ear bars were replaced by a hollow speculum that accommodated a sound delivery system.

### Acoustic stimuli and electrophysiological recording

Extracellular single unit responses were recorded from neurons in the IC and CN in two separate sets of experiments. For the IC recordings, a craniotomy was performed to expose the cerebral cortex overlying the IC and a tungsten electrode (1 – 2 MΩ) (Merrill and Ainsworth, 1972) was lowered through the cortex by means of a piezoelectric microdrive (Burleigh 6000 ULN). Neuron identification in the IC was based on stereotaxic coordinates, physiological criteria of tonotopicity, and response properties (Rees et al., 1997; LeBeau et al., 2001; Malmierca et al., 2003; Hernández et al., 2005; Pérez-González et al., 2005, 2006). An electrode dorsoventral penetration (with an angle of 20° from the frontal plane) through the central nucleus of the IC is identified by the stepwise progression from low to high frequencies (Malmierca et al., 2008) and by the constant presence of tonically firing units (Rees et al., 1997). Typical responses of the neurons in the cortices of the IC (i.e., dorsal, lateral, and rostral) are characterized by longer response latencies, predominantly on-phasic firing patterns and weaker tonic firing than those from the central nucleus. Cortical IC neurons commonly display broadly tuned, W-shaped, or other complex tuning curves (Lumani and Zhang, 2010; Geis et al., 2011; Duque et al., 2012) and a clear topographic organization of the frequencies along the dorsal cortex is not present (Malmierca et al., 2008; Lumani and Zhang, 2010). For the recording of CN neurons, part of the cerebellum was carefully aspirated to visually localize the dorsal cochlear nucleus (DCN). Glass micropipettes filled with 2 M NaCl (15 – 25 MΩ) or tungsten electrodes (1 – 2 MΩ) were advanced into the DCN. For some IC experiments and most of the CN recordings, an electrolytic lesion (10 – 15 μA for 10 – 15 s) was applied for subsequent histological verification of the recording site. Brains were fixed using a mixture of 1% paraformaldehyde and 1% glutaraldehyde diluted in 0.4 M phosphate buffer saline (0.5% NaNO_3_ in PBS). After fixation, tissue was cryoprotected in 30% sucrose and sectioned in the coronal or sagital plane at a thickness of 40 μm on a freezing microtome. Slices were Nissl stained with 0.1% cresyl violet to facilitate identification of cytoarchitectural boundaries. The CN units were assigned to one of the two main divisions (dorsal or ventral) of the nucleus using as reference the standard sections from a rat brain atlas (Paxinos and Watson, 2007).

Acoustic stimuli were delivered through a sealed acoustic system (Rees, 1990; Rees et al., 1997) using two electrostatic loudspeakers (TDT-EC1) driven by two TDT-ED1 modules. Search stimuli were pure tones or noise bursts monaurally delivered under computer control using TDT System 2 hardware (Tucker-Davis Technologies) and custom software (Faure et al., 2003; Pérez-González et al., 2005, 2006; Malmierca et al., 2008). The output of the system at each ear was calibrated *in situ* using a ¼ inch condenser microphone (Brüel and Kjær 4136, Nærum, Denmark) and a DI-2200 spectrum analyzer (Diagnostic Instruments Ltd., Livingston, Scotland, UK). The maximum output of the TDT system was flat from 0.3 to 5 kHz (~100 ± 7 dB SPL) and from 5 to 40 kHz (90 ± 5 dB SPL). The highest frequency produced by this system was limited to 40 kHz. The second and third harmonic components in the signal were 40 dB or more below the level of the fundamental at the highest output level (Hernández et al., 2005; Malmierca et al., 2009).

Action potentials were recorded with a BIOAMP amplifier (TDT), the 10× output of which was further amplified and bandpass-filtered (TDT PC1; *f*_*c*_: 0.5 – 3 kHz) before passing through a spike discriminator (TDT SD1). Spike times were logged at one microsecond resolution on a computer by feeding the output of the spike discriminator into an event timer (TDT ET1) synchronized to a timing generator (TDT TG6). Stimulus generation and on-line data visualization were controlled with custom software. Spike times were displayed as dot rasters sorted by the acoustic parameter varied during testing.

Once a neuron was isolated, the monoaural frequency response area (FRA), i.e., the combination of frequencies and intensities capable of evoking a response, was obtained by an automated procedure with 5 stimulus repetitions at each frequency (from 0.5 to 40 kHz, in 20 – 30 logarithmic steps) and intensity step (steps of 10 dB) presented randomly at a repetition rate of 4 Hz. The stimuli used to generate the tuning curves were pure tones with duration of 75 ms. The neuronal response to the combination of frequencies and intensities was plotted using MATLAB software (Mathworks, Inc.) and the best frequency (BF) and threshold for each neuron were identified.

### Stimulus presentation paradigms

**Figure 1:**
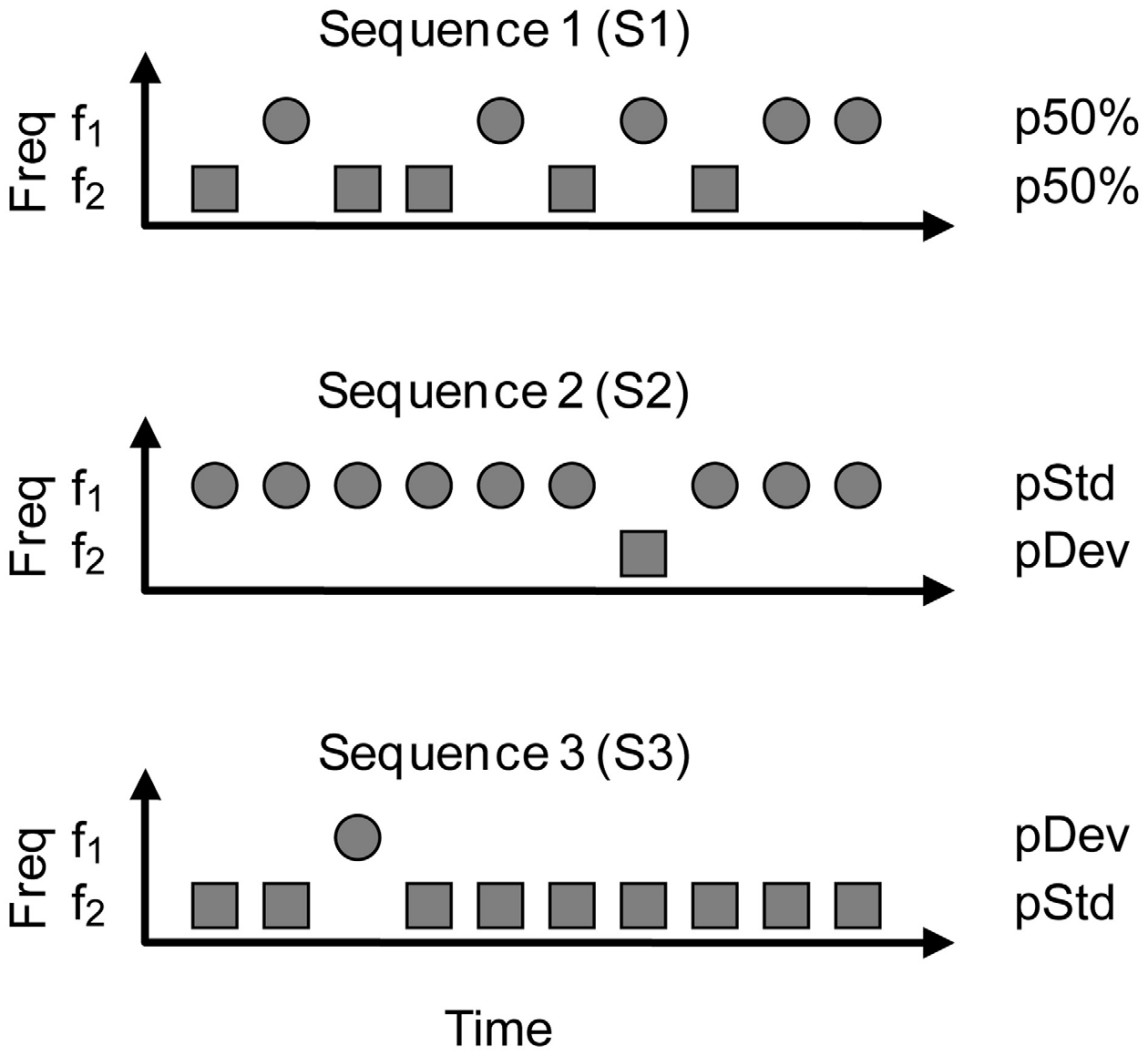
**The oddball stimulation paradigm.** Two frequencies (*f*_1_, *f*_2_) were presented pseudo-randomly with different probabilities of occurrence. In Sequence 1 (S1), *f*_1_, and *f*_2_ occurred with equal probability (p50%), which served as a control condition. This condition is useful to see the neuron's tuning to the frequencies chosen. For the oddball condition, the probability of the frequencies was modified such that one frequency (*f*_1_, circles) was the standard tone, occurring with high probability, and the other (*f*_2_, squares) was the deviant tone, with low probability of occurrence (Sequence 2, S2). The probabilities of *f*_1_ and *f*_2_ were reversed in Sequence 3 (S3) in order to have each frequency presented as deviant and standard. We tested two probabilities for the deviant tone (pDev), 30 and 10%, so the corresponding probabilities for the standard (pStd) were 70 and 90%, respectively.

For all neurons, stimuli were presented in an oddball paradigm similar to that used to record mismatch negativity responses in human studies (Näätänen, 1992), and more recently in the cat auditory cortex (Ulanovsky et al., 2003, 2004), rat IC (Malmierca et al., 2009; Pérez-González et al., 2012) and auditory thalamus (Antunes et al., 2010; Antunes and Malmierca, 2011). Briefly, we presented two stimuli consisting of pure tones at two different frequencies (*f*_1_ and *f*_2_), that elicited a similar firing rate and response pattern at the same level of 10 – 40 dB SPL above threshold. Both frequencies were within the excitatory response area previously determined for the neuron. A train of 400 stimulus presentations containing both frequencies was delivered in three different sequences (Figure 1). The repetition rate of the train of stimuli for the IC neurons was 4 Hz, as it has been previously demonstrated to be suitable to elicit SSA in IC neurons of the rat (Malmierca et al., 2009). In the CN recordings, we explored repetitions rates of 4, 8, 12, and 20 Hz. Due to the different repetition rates used, the duration of the pure tones was 75 ms for the IC recordings (Hernández et al., 2005) and 25 ms for the CN recordings (in a few recordings at 4 and 8 Hz, tones lasted 75 ms as well), including a 5 ms rise/fall ramp for both cases.

As shown in Figure 1, in Sequence 1 (S1) both frequencies were presented with the same probability of occurrence (equiprobable condition; *p*(*f*_1_) = *p*(*f*_2_) = 50%). In Sequence 2 (S2), one frequency (*f*_1_) was presented as the standard (i.e., high probability within the sequence: 90 or 70%); interspersed randomly among the standards were the deviant stimuli (i.e., low probability: 10 or 30%, respectively) at the second frequency (*f*_2_). After obtaining one data set, the relative probabilities of the two stimuli were reversed, with *f*_2_ as the standard and *f*_1_ as the deviant (S3). Sequences 2 and 3 constitute what we refer to as an oddball condition. The responses to the standard and deviant stimuli were normalized to spikes per stimulus, to account for the different number of presentations in each condition, because of the different probabilities. We tested several frequency separations between *f*_1_ and *f*_2_, expressed as frequency contrast Δ*f* = (*f*_2_ − *f*_1_)/(*f*_2_ × *f*_1_)^1/2^ (Ulanovsky et al., 2003). As the frequency pairs were chosen to evoke similar firing rates in responses to both tones, Δ*f* ranged from 0.02 to 3. The Δ*f* values were grouped into three intervals: Δ*f* ≤ 0.07, 0.07< Δ*f* ≤ 0.2 and Δ*f* > 0.2 (≤ 0.101, 0.101< Δ*f* ≤ 0.288 and Δ*f* > 0.288 octaves, respectively), in order to approximate to the values used in other studies, i.e., Δ*f* = 0.04, 0.10, and 0.37 (Ulanovsky et al., 2003, 2004; Malmierca et al., 2009). The same paradigm was repeated changing the probability of the deviant tone (pDev = 10%, 30%) or the Δ*f*. For the CN experiments, we only tested pDev = 10% and Δ*f* = 0.1. The presentation of sequences at different deviant probabilities and at different repetition rates was randomized.

### Data analysis

We measured the sharpness of the FRA of IC neurons calculating the bandwidth (BW) and Q-values at 10 and 40 dB SPL above the threshold as in our previous work (Hernández et al., 2005; Malmierca et al., 2009). The BW at *n* dB expresses the difference in kHz between the lower (F_L_) and upper (F_U_) frequencies of the FRA (BW_*n*_ = *F*_U_ − *F*_L_). The Q-value is calculated as the characteristic frequency (CF) divided by the BW at *n* dB above threshold (Q_*n*_ = CF/BW).

The amount of SSA was quantified by two indices that have been used in previous studies (Ulanovsky et al., 2003, 2004; Malmierca et al., 2009; Antunes et al., 2010; Antunes and Malmierca, 2011; Pérez-González et al., 2012). The first index was the Frequency-Specific SSA Index (SI) defined as: SI(*f*_*i*_) = [d(*f*_*i*_) − s(*f*_*i*_)]/[d(*f*_*i*_) + *s*(*f*_*i*_)], where *i* = 1 or 2 and *d*(*f*_*i*_) and *s*(*f*_*i*_) are responses (as normalized spike counts) to frequency *f*_*i*_ when it was deviant or standard, respectively. The second one was the Common-SSA Index (CSI) defined as CSI = [*d*(*f*_1_) + d(*f*_2_) − *s*(*f*_1_) − s(*f*_2_)]/[*d*(*f*_1_) + *d*(*f*_2_) + *s*(*f*_1_) + *s*(*f*_2_)], where *d(f)* and *s(f)* are responses to each frequency *f*_1_ or *f*_2_ when they were the deviant (*d*) or standard (*s*) stimulus, respectively. These indices reflect the extent to which the neuron responds more strongly to the frequencies when they are deviant compared to when they are standard. The possible SI and CSI values range from −1 to +1, being positive if the response to the deviant stimulus is greater and negative if the response to the standard stimulus is greater.

To estimate the neuronal sensitivity we performed a receiver operating characteristic (ROC) analysis (Tanner and Swets, 1954; Cohn et al., 1975; Fawcett, 2006; for a review of the use of ROC in psychometric and neurometric data analysis, see Stüttgen et al., 2011). This analysis has been previously used to measure the ability of CN units to signal changes in intensity (Shofner and Dye, 1989) and the sensitivity of IC units to interaural-time differences and binaural correlation (Skottun et al., 2001; Shackleton et al., 2003, 2005; Gordon et al., 2008). It is assumed that when different stimuli elicit different firing rates the response of a neuron provides the basis for discriminating between them. However, there is also a substantial variability in the responses to each stimulus, so the distributions of firing rates to similar stimuli overlap, and thus discrimination based upon firing rate will only be correct on a proportion of trials. The ROC analysis allows us to calculate the performance of the best possible discriminator between the two frequencies which is based on spike counts only. This discriminator is a function of the two probability distributions of spike counts in response to the two stimuli.

The ROC plots the probability of correct detection of *f*_2_ against the probability of “false alarm” detection of *f*_2_ when *f*_1_ occurred. Since detection is assumed to be based on spike counts only, trials have to be classified to one or the other frequency based solely on the evoked spike count. Thus, any discriminator between the two frequencies consists, in practice, of a list of spike counts that are assigned to frequency *f*_1_, with all other spike counts assigned to frequency *f*_2_ (we do not need to consider so-called “randomized rules” here, because we are only interested in the integral of the ROC, see below). In many studies, ROCs are calculated by a threshold on spike counts: all spike counts below the threshold are assigned to one frequency, and those above the threshold to the other. However, the lemma of Neyman and Pearson (Maris, 2012) requires spike counts to be assigned to frequencies based on their likelihoods, the ratio *p*(*n*|*f*_2_)/*p*(*n*|*f*_1_). For an optimal decision rule, a threshold is selected, and all spike counts whose likelihood is larger than that threshold are assigned to *f*_2_ (with the others assigned to *f*_1_). The probabilities of correct decision and false alarm for this decision rule can then be calculated in a straightforward manner. The ROC is obtained by calculating these probabilities while varying the threshold.

Then, we calculated the area under the ROC curve (AUC) as an estimate of the neural discriminability of frequency. The AUC corresponds to the probability of correct stimulus detection expected from an ideal observer in a two-alternative forced-choice psychophysical task (Green and Swets, 1966; Fawcett, 2006). Thus, sensitivity measured as AUC varies between 0.5 and 1, where 0.5 occurs when the spike count distributions for frequencies *f*_1_ and *f*_2_ are identical, and 1 indicates complete separation of the distributions. To compensate for sampling bias, we corrected each AUC value by performing 10,000 permutations of the original spike count distributions, assigned randomly to either *f*_1_ or *f*_2_, calculated the corresponding AUCs, and subtracted their mean value from the original AUC. Due to this correction some of the AUC values we report are smaller than 0.5. We also used the permutations test to estimate the probability of the AUC being significantly larger than 0.5. This way, we obtained one AUC value for the equiprobable condition (S1) and two AUC values for the oddball conditions (S2, S3). We used the mean AUC of S2 and S3 for the analyses instead of the maximum value as in previous works (Ulanovsky et al., 2003; Malmierca et al., 2009), in order to avoid an upward bias.

The CSI values were tested against zero by bootstrapping (1000 samples) in order to estimate a 95% confidence interval. Typically, CSI values smaller than 0.1 were not statistically different from zero (85% of all cases with CSI < 0.1 and 15% of the cases with CSI > 0.1). Thus, CSI values within the range of −0.1 to 0.1 were considered be due to random fluctuations in spike counts. This procedure provided a CSI cutoff comparable to other values previously set with different criteria (e.g., CSI = 0.18 for auditory thalamus of the rat; Antunes et al., 2010). It may be somewhat smaller than the cutoff in thalamus because of the lower variability in the responses of IC neurons (e.g., Chechik et al., 2006).

## Results

To investigate how frequency sensitivity is affected by the stimulation context we recorded the response of 224 well isolated single units in the IC and 51 units in the CN using an oddball paradigm. The frequency contrast (Δ*f* ≤ 0.07, 0.07< Δ*f* ≤ 0.2, Δ*f* > 0.2) and probability of the deviant tone (pDev = 30% or 10%) were varied in IC recordings, and the repetition rate (4, 8, 12, and 20 Hz) in the CN. Additionally, an equiprobable context (*p*(*f*_1_) = *p*(*f*_2_) = 50%) was tested as control condition in both sets of experiments.

### Neurons in the IC show different degrees of SSA and stimulus discriminability

**Figure 2:**
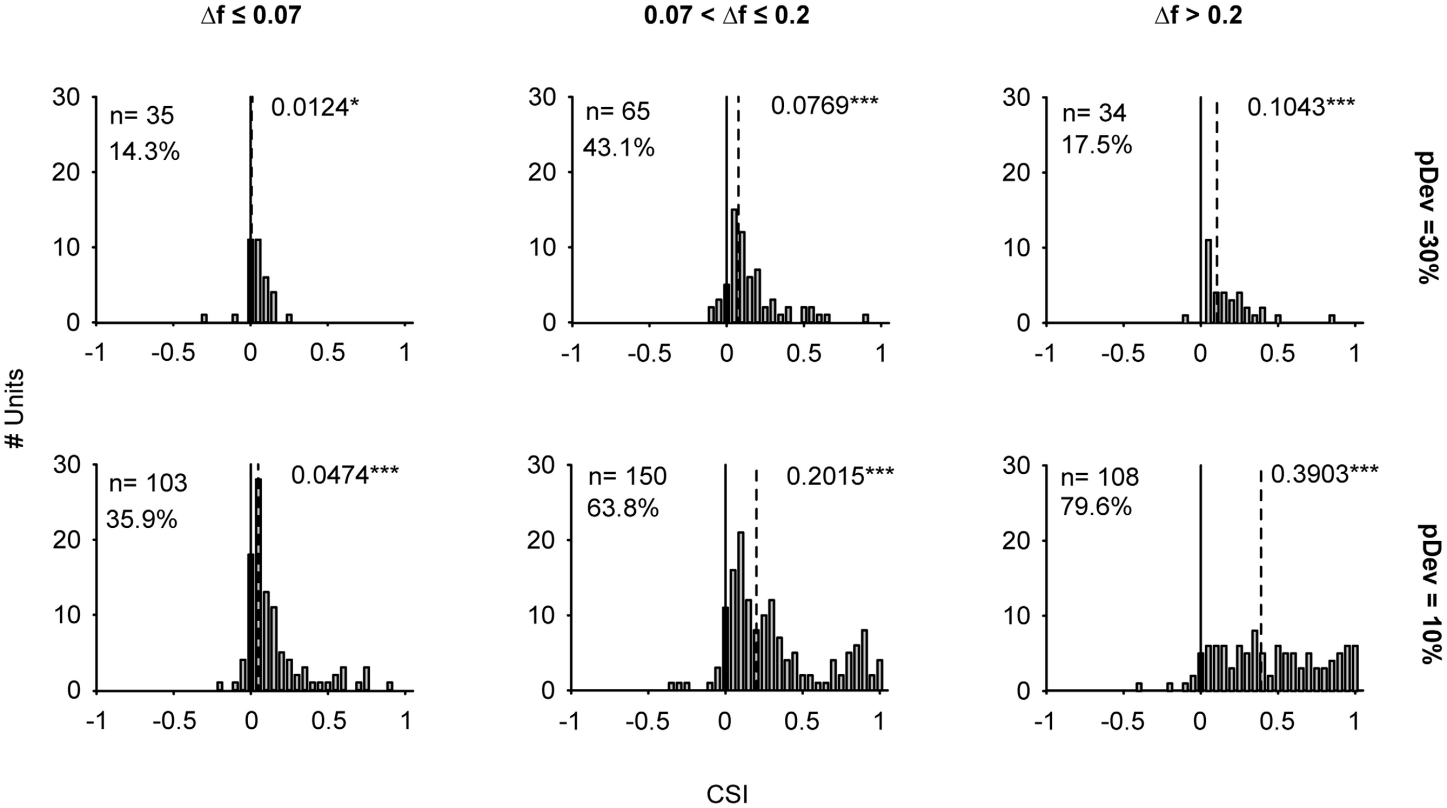
**Distribution of stimulus-specific adaptation indices of IC neurons.** Histograms of the common SSA index (CSI) displayed according to the frequency separation intervals (columns: Δ*f* ≤ 0.07, 0.07 < Δ*f* ≤ 0.2, Δ*f* > 0.2) and the probability of the deviant tone (rows: pDev = 30 and 10%). The CSI was calculated from the responses recorded in S2 and S3. A CSI = 0 indicates an equal neuronal response when the tones were presented as deviant or standard, while positive and negative values represent higher firing rates when the tones were deviant or standard, respectively. For each stimulus condition the CSI values were tested against zero (solid line). The numbers next to the dashed line indicate the value of the median and the statistical significance (Signed Rank Test; ^*^p < 0.05, ^***^p < 0.001). The distributions moved toward positive values when Δ*f* was larger and pDev smaller. The percentages indicate the amount of neurons with CSI > 0.1.

As might be expected from our previous studies (Pérez-González et al., 2005; Malmierca et al., 2009), neurons in the IC exhibited different degrees of SSA. Figure 2 shows the distribution of the CSI under different stimulus conditions in the current sample. The distributions of CSI are skewed toward positive values, and their medians are significantly different from zero (Signed Rank Test; *p* < 0.05) regardless of the condition tested (Figure 2). Positive CSI values reflect a stronger response to the deviant tone than to the standard one. The effects of frequency separation and deviant probability were tested using a Two-Way ANOVA on Δ*f* × probability. There was a main effect of Δ*f* (*F*_(2, 489)_ = 18, *p* = 0) and of probability condition [*F*_(1, 489)_ = 39, *p* = 0]. The interaction just failed to reach significance [*F*_(2, 489)_ = 2.5, *p* = 0.08]. *Post-hoc* comparisons showed that the most positive CSI values were observed for deviant probability of 10% at the two highest frequency contrast intervals; 0.07< Δ*f* ≤ 0.2 and Δ*f* > 0.2. For the 10% probability condition, the CSIs increased significantly with increased frequency separation: CSI_10%/Δ*f* > 0.2_ > CSI_10%/0.07 < Δ*f* ≤ 0.2_ > CSI_10%/Δ*f* ≤ 0.07_. On the other hand, the *post-hoc* comparisons did not show a significant difference between the average CSIs in the 30% condition and different frequency separations. There was also a significant difference due to changes in deviant probability for the two highest frequency separation intervals: CSI_10%/0.07 < Δ*f* ≤ 0.2_ > CSI_30%/0.07 < Δ*f* ≤ 0.2_; CSI_10%/Δ*f* > 0.2_ > CSI_30%/Δ*f* > 0.2_. This trend was emphasized by the higher percentage of neurons with CSI values larger than 0.1 when deviant probability was 10% compared to 30% (percentages indicated in Figure 2). From the six groups, only seven neurons (3.1%) showed CSI ≤ −0.1.

**Figure 3:**
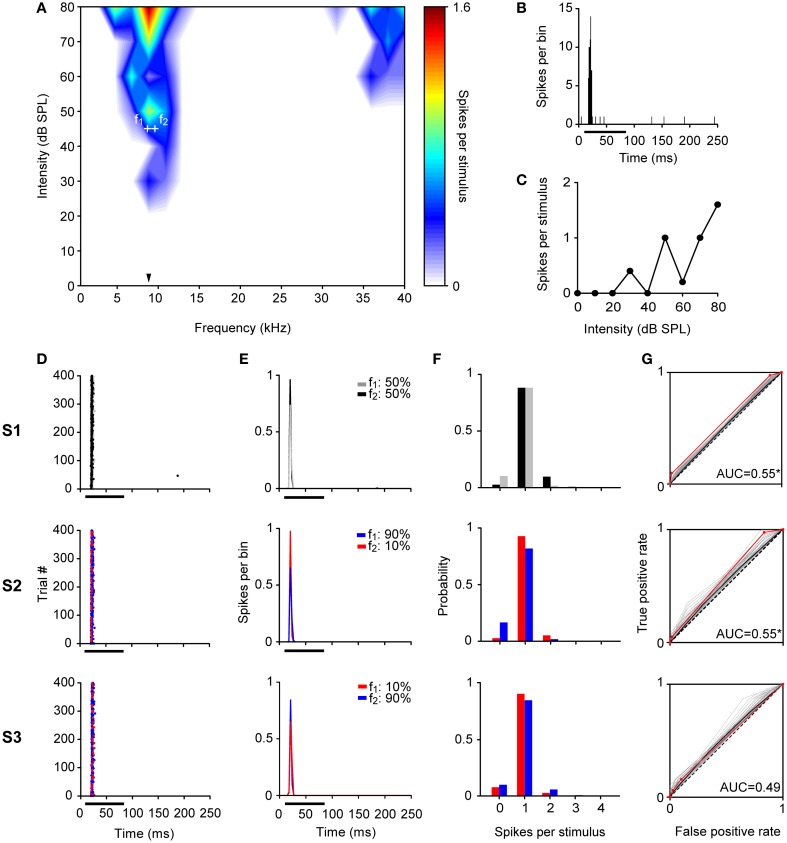
**Example of a non-adapting neuron in the IC. (A)** Narrow FRA in color code for response magnitude. The tested frequencies (*f*_1_: 8.7 kHz, *f*_2_: 9.6 kHz, white crosses) were chosen around the BF (8.8 kHz) (arrowhead), at 45 dB SPL. **(B)** PSTH of the accumulated response to all the frequencies (0.5 – 40 kHz) and intensities (0 – 80 dB SPL) presented (1 ms bins). **(C)** Rate-level function at BF. **(D–G)** The responses of the neuron for each pair of stimuli for each of the three sequences (S1, S2, S3) are shown as dot raster plots **(D)**, PSTH (3 ms bins) **(E)**, spike probability distributions **(F)**, and ROC curves **(G)**. In the dot raster each dot represents the occurrence of a spike. The black bar under the PSTH and dot raster plots indicates the duration of the stimulus (75 ms). The probability of each frequency for each sequence is indicated on the upper left of the **(E)** panels. In the ROC curves **(G)** the dashed line corresponds to random guessing or no stimulus discrimination (AUC = 0.5), indicating complete overlap of the spike probability distributions. The red line represents the ROC curve calculated using the recorded data, the curves plotted in gray were obtained with the permutation method of the original spike count distributions, and the black line is represents the mean ROC curve of permutations. A total of 10,000 permutations were calculated, but for visual clarity only 100 curves are displayed. For each ROC curve, the area under the ROC curve (AUC) is shown corresponding to the original AUC value minus the mean AUC from permutations, as well as, the significance value for AUC > 0.5 (Permutation test; ^*^p < 0.05). The repetition rate was 4 Hz and the frequency separation was 0.141 octaves. This neuron did not show SSA (CSI = 0.04, Bootstrapping; *p* > 0.05), displaying a very similar response to *f*_1_ and *f*_2_ across the three sequences regardless the probability of each tone.

**Figure 4:**
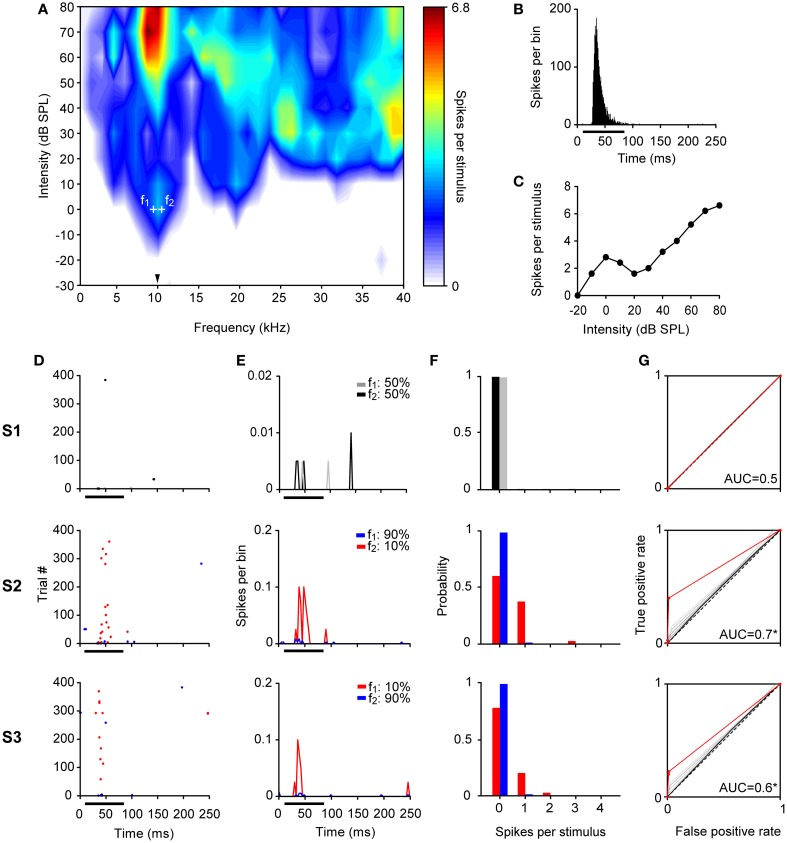
**Example of an adapting neuron in the IC. (A)** FRA of a broadly tuned neuron with a BF of 10 kHz (arrowhead). The frequencies tested are indicated by the white crosses around the BF (*f*_1_: 9.5 kHz, *f*_2_: 9.6 kHz), at 0 dB SPL. **(B–G)** Same format as in Figure 3. This neuron showed strong SSA (CSI = 0.88, Bootstrapping; *p* < 0.05) reducing its firing to the high probability tone in S2 and S3 while still responding to the low probability one across most stimulus presentations. This differential firing is reflected in an AUC larger than 0.5 (Permutation test; ^*^p < 0.05) in oddball sequences (S2 and S3).

**Figure 5:**
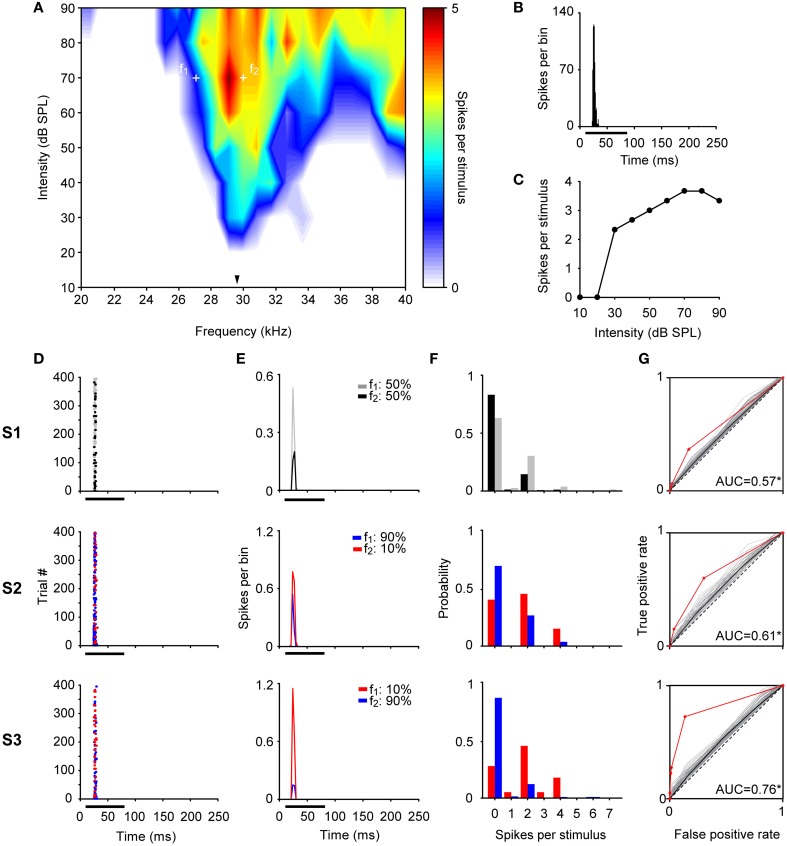
**Example of a partially-adapting neuron in the IC. (A)** FRA from a neuron with a BF of 29.9 kHz (arrowhead). The frequencies tested were *f*_1_: 27.1 kHz and *f*_2_: 30 kHz, at 70 dB SPL. **(B–G)** Same format as in Figure 3. This neuron displayed a significant level of SSA (CSI = 0.5, Bootstrapping; *p* < 0.05), responding to both tones across the 400 stimulus trials. Although, the neuron displayed significant discriminability in the equiprobable condition (S1, AUC = 0.57) (Permutation test; ^*^p < 0.05), this was improved under the oddball sequences (S2, AUC = 0.61; S3, AUC = 0.76).

Examples of individual IC neurons exhibiting different CSI values are shown in Figures 3–5. The deviant probability for the three cases was 10% and the frequencies tested (*f*_1_, *f*_2_) in these examples were separated by 0.144 octaves (Δ*f* = 0.1) around its BF at an intensity of 10 – 50 dB above threshold.

Figure 3 illustrates a neuron with a CSI not significantly different from zero (CSI = 0.04; *p* > 0.05). This neuron had a narrow FRA with a narrow bandwidth both at 10 and 40 dB SPL above its threshold (BF = 8.8 kHz, *Q*_10_ = 5.62 and *Q*_40_ = 1.22) (Figure 3A). It had an onset firing pattern (Figure 3B) and showed a mixed/complex rate-level function (Figure 3C). The responses elicited in the equiprobable condition (S1) and oddball condition (S2 and S3) are shown as dot rasters (Figure 3D) as well as the peristimulus time histograms (PSTH) (Figure 3E). Figure 3F displays the corresponding spike-count distributions and the ROC curve is shown in Figure 3G. This neuron displayed a very robust and reliable response across the 400 stimulus presentations. In consequence, its spike count distributions were very different from Poisson distributions: while the average spike count is about 1, the probability of having zero spike counts is much smaller than that of either frequency evoking a single spike (for a Poisson distribution, these two probabilities should be approximately equal when the mean spike count is close to 1). The spike-count distributions for *f*_1_ and *f*_2_ were very similar, overlapping almost completely (Figure 3F), although the average spike count was slightly larger for *f*_2_ than for *f*_1_. The large overlap between these distributions resulted in AUC values very close to 0.5, but the very low variability resulted in an AUC that was significantly larger than 0.5 in the equiprobable condition. When *f*_2_ was the deviant, this difference was maintained, but when *f*_1_ was the deviant, the average spike count in response to *f*_2_ decreased slightly, enough to render the AUC not significantly different from 0.5 (AUC(S1) = 0.55, *p* = 0; AUC(S2) = 0.55, *p* = 0.01; AUC(S3) = 0.49, *p* = 0.65). Thus, the frequency discrimination capability of this neuron was poor in an equiprobable context and did not improve in an oddball stimulation context, consistent with its low CSI.

By contrast, neurons with high CSI values fired significantly differently in response to deviant and standard tones in the oddball condition. The neuron illustrated in Figure 4 had a CSI = 0.88 (*p* < 0.05). It was tuned to a wide range of frequencies (Figure 4A) reflected by its low Q-values (BF = 10 kHz, *Q*_10_ = 0.74 and *Q*_40_ = 0.27). This neuron also had an onset firing pattern, although it showed a large variability of first spike latency (FSL) (Figure 4B) and had a non-monotonic rate-level function (Figure 4C). During the equiprobable presentation of the tones (S1), this neuron adapted its response to both frequencies, and had a very low probability to respond at all (*P*_≥1*sps*_ = 0.005). In the oddball condition, responses to the standard tone remained extremely sparse, but deviant trials did evoke a few spikes with higher probability. Thus, the overlap between the spike-count distributions was reduced substantially (probability of firing ≥ 1 sps in response to the deviant/standard was 0.4/0.017 and 0.23/0.014 for S2 and S3, respectively). As a result, the AUCs for the oddball conditions were higher than for the equiprobable condition [AUC(S1) = 0.5, *p* = 0.5; AUC(S2) = 0.7, *p* = 0; AUC(S3) = 0.6, *p* = 0].

The examples shown in Figures 3 and 4 are extreme cases, and neurons in the IC showed a continuous distribution of SSA as depicted in Figure 2. For example, Figure 5 illustrates a partially-adapting neuron (CSI = 0.5; *p* < 0.05) tuned to high frequencies (Figure 5A) and with a non-monotonic rate-level function (Figure 5C). The bandwidth of the FRA increased between 10 and 40 dB above threshold, respectively (BF = 29.9 kHz, *Q*_10_ = 7.81 and *Q*_40_ = 2.25). This neuron showed a poor, although significant discrimination capability at the equiprobable condition [AUC(S1) = 0.57, *p* = 0] which improved in the oddball condition [AUC(S2) = 0.61, *p* = 0; AUC(S3) = 0.76, *p* = 0].

### Frequency discriminability depends on stimulus context in the IC

**Figure 6:**
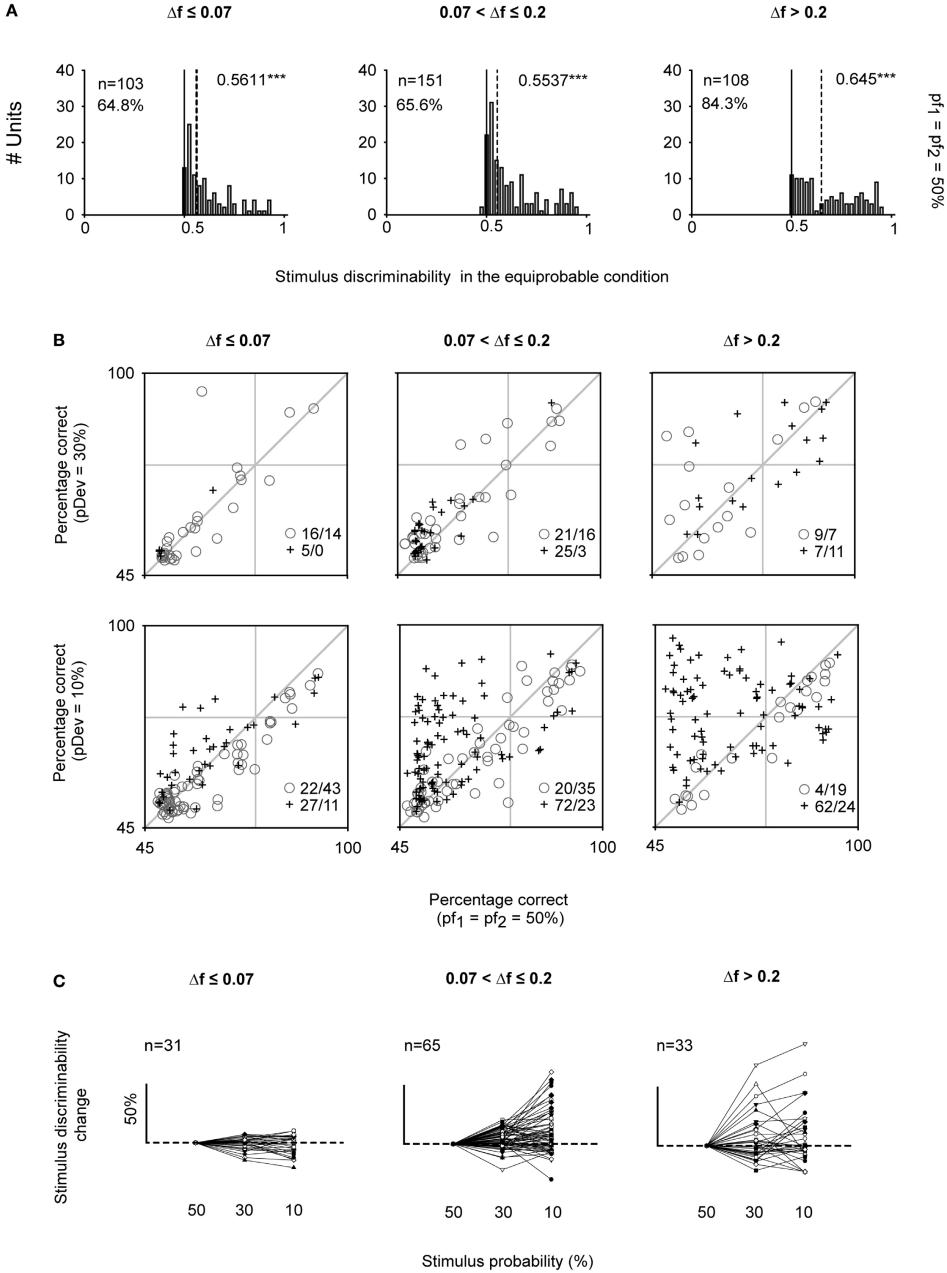
**Neurometric performance under equiprobable and oddball conditions of IC neurons. (A)** Distributions of the AUC values for the equiprobable condition (AUC_50%_) indicating the median (dashed line) significantly differs from 0.5 (Signed Rank Test; ^***^p < 0.001). The percentage of neurons whose AUC_50%_ was higher than 0.5 is indicated for each panel (Permutation test; *p* < 0.05). **(B)** Scatter plots showing the neurometric performance for frequency discrimination expressed as percentage correct under the oddball condition (rows: pDev = 30, 10%) versus the equiprobable condition (*pf*_1_ = *pf*_2_ = 50%), for each frequency contrast interval (columns: Δ*f* ≤ 0.07, 0.07 < Δ*f* ≤ 0.2, Δ*f* > 0.2). Separately are represented the neurons with CSI ≤ 0.1 (gray circles) and CSI > 0.1 (dark crosses). For the oddball condition, the percentage correct corresponds to the mean AUC value obtained from S2 and S3. The number of neurons above and below the diagonal line (equal performance in both conditions) is indicated by the inset on the bottom right of each panel. **(C)** Sensitivity curves of individual neurons expressed as percentage of change elicited when pDev = 10% and 30% regarding the *pf*_1_ = *pf*_2_ = 50% condition.

IC neurons were able to discriminate very similar frequencies even when both tones had the same probability of occurrence (*p*(*f*_1_) = *p*(*f*_2_) = 50%). The tested frequencies were selected online to evoke similar response magnitudes. Nevertheless, the noise in the estimation of response rates resulted in some imbalance between the responses to the two frequencies, leading to significant discriminability between them. The discriminability elicited under the equiprobable condition (AUC_50%_) across the three Δ*f* intervals significantly differed from a mere random discrimination (AUC = 0.5, Signed Rank Test; *p* < 0.001) (Figure 6A). Furthermore, more than half of the neurons from each frequency separation group had AUC_50%_ significantly larger than 0.5 (*p* < 0.05) (Figure 6A, indicated in percentage). In a substantial number of cases, AUC_50%_ exceeded 0.71 (24.1, 23.2, and 41.6% for the three Δ*f* groups), the generally accepted definition of a threshold (Green and Swets, 1966). Neurons with AUCs above this threshold for the smallest frequency contrast interval (Δ*f* ≤ 0.07) had narrower bandwidths (*Q*_10_ = 6.23 ± 5.43) that the rest of neurons (*Q*_10_ = 3.53 ± 5.47) (Signed Rank Test; *p* < 0.05).

To address the central question of this paper, Figure 6B compares the percent correct (as estimated by AUC) in the oddball and equiprobable condition for each neuron. For the oddball condition, we used the mean discriminability (AUC_oddball_) from the values elicited in the two oddball sequences since there was not significant difference in the AUC values elicited in S2 and S3 (Rank Sum Test; *p* > 0.05). Neurons whose discriminability was unaffected in the oddball condition fell along the diagonal line. Neurons under the diagonal line showed a better discriminability in the equiprobable condition. By contrast, neurons that improved their discriminability in the oddball paradigm were located above the diagonal. Neurons with CSI > 0.1 are marked by crosses, the others are marked by circles. When pDev = 10%, there was a larger proportion of neurons with CSI > 0.1 than neurons with CSI ≤ 0.1 that showed improved discriminability in the oddball condition (χ^2^ = 58.6, df = 1, *p* < 0.001), but these proportions did not depend on frequency separation (χ ^2^ = 5.4, df = 2, *p* = 0.07). For this probability condition, the AUCs of neurons with CSI ≤ 0.1 were slightly, although significantly, smaller in the oddball than in the equiprobable condition (^46^/_96_, neurons above and below the bisecting line, respectively, for all frequency separation classes together). This effect was due presumably to the poorer sampling of the spike count histograms for the deviant stimuli in the oddball condition. On the other hand, AUC_oddball_ increased substantially for neurons with CSI > 0.1 (^161^/_59_ neurons above and below the bissecting line, respectively). The increase resulted in many neurons whose frequency discrimination was below threshold in the equiprobable condition (AUC_50%_ < 0.71) and that exceed threshold in the oddball conditions (AUC_oddball_ > 0.71). Within this subset of neurons, there are cases in which the neurometric performance reached values close to 100% correct in the oddball condition. Such cases were much more common at the largest frequency contrasts (0.07< Δ*f* ≤ 0.2 and Δ*f* > 0.2). For pDev = 30%, the discriminability did not change consistently relative to the equiprobable condition, and proportions of neurons with slight increase or decrease in discriminability were as common in the different frequency difference classes (χ^2^ = 5.4, df = 2, *p* = 0.07) and among CSI classes (χ^2^ = 3.9, df = 1, *p* = 0.05).

In order to verify whether the same trend was observed at the level of single neurons, we obtained the individual “sensitivity curves” for the neurons that were tested under all probabilities conditions (50, 30, and 10%) and for the same frequency pairs (Figure 6C). The discriminability increment was expressed as the percentage of change in AUC_oddball_ relative to the discriminability displayed under the equiprobable condition (AUC_50%_). These sensitivity curves revealed a considerable diversity in the neuronal performance. Both neuron identity and stimulus probability had a significant effect on the discrimination capability for the intermediate Δ*f* interval [Two-Way ANOVA on stimulus probability × neuron, significant main effect of stimulus probability: *F*_(2, 128)_ = 7.7, *p* < 0.001], but for the smallest and largest Δ*f* the main effect of stimulus probability was not significant.

**Figure 7:**
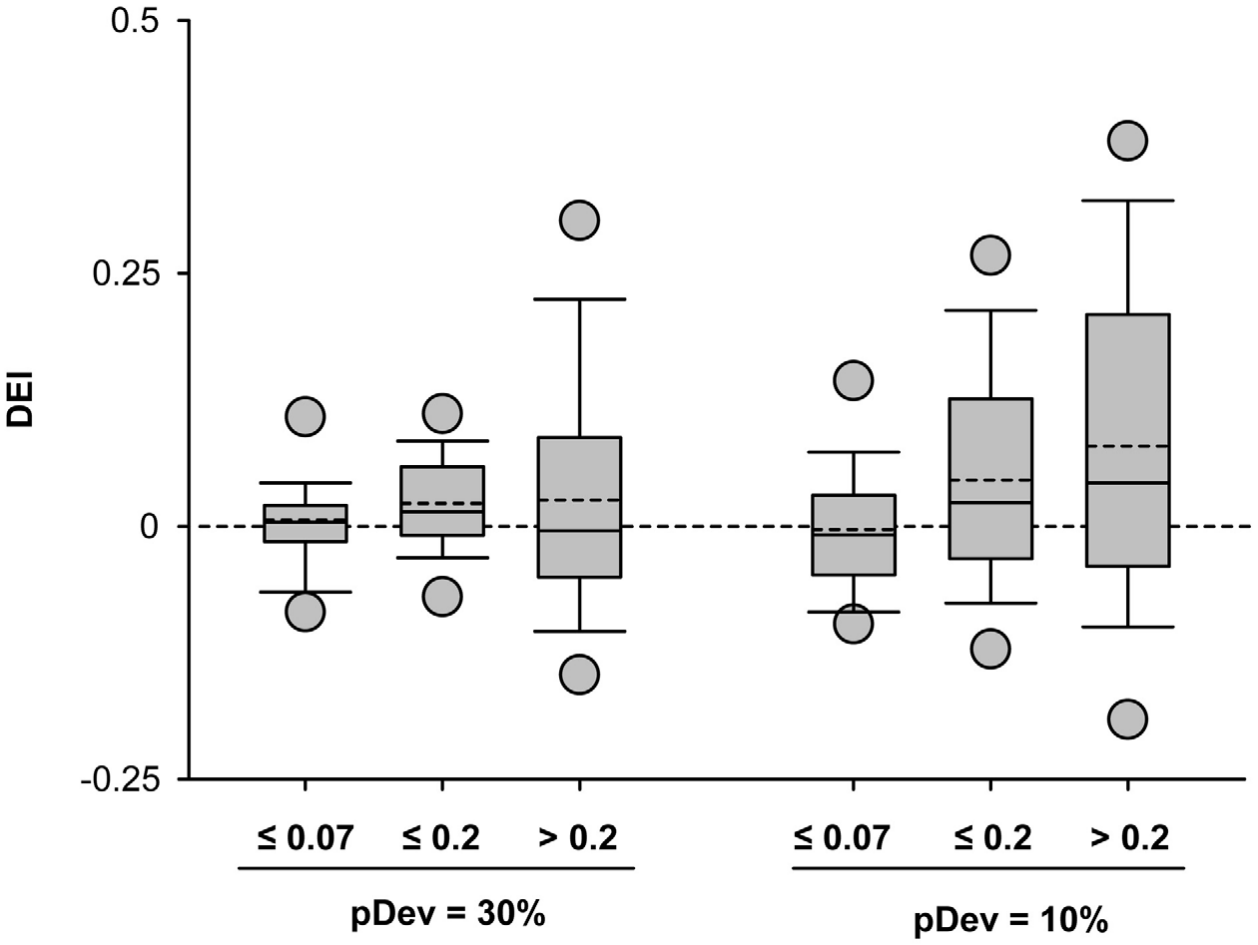
**Stimulus discriminability enhancement of IC neurons across different stimulus conditions.** Box plots of the discriminability enhancement under the oddball condition (DEI) showing the mean (dashed line) and the median (solid line) values, as well as, the 5^th^ and 95^th^ outliers. All the mean values were positive (except for the ^10%^/Δ*f* ≤ 0.07 condition), reflecting a better stimulus discrimination when one of the frequencies is presented as a deviant tone, that is, with low probability of ocurrence (30 or 10%). The DEIs were only affected by the frequency separation [*F*_(2, 489)_ = 5.72, *p* < 0.01] (Two-Way ANOVA, deviant probability × frequency separation).

Since some neurons under the equiprobable condition showed significant discriminability values that exceeded a mere random response (Figure 6A), we took into account this neuron-specific tuning. We calculated the *discriminability enhancement index* (DEI) as the difference between the discriminability elicited in the oddball condition and that elicited in the equiprobable one (DEI = AUC_oddball_ − AUC_50%_). DEI ranges from −0.5 to 0.5, with positive values indicating an improvement in discriminating two stimuli under an oddball context. The comparison of the mean population values of DEI across all stimulus combinations (Two-Way ANOVA, stimulus probability × Δ*f*) demonstrated that it was affected by the frequency separation [*F*_(2, 489)_ = 5.72, *p* < 0.01] but not by stimulus probability [*F*_(1, 489)_ = 3.71, *p* = 0.055], with no interaction between those factors [*F*_(2, 489)_ = 2.1, *p* = 0.12] (Figure 7).

### IC neurons with high SSA showed a greater discriminability enhancement under oddball conditions

**Figure 8:**
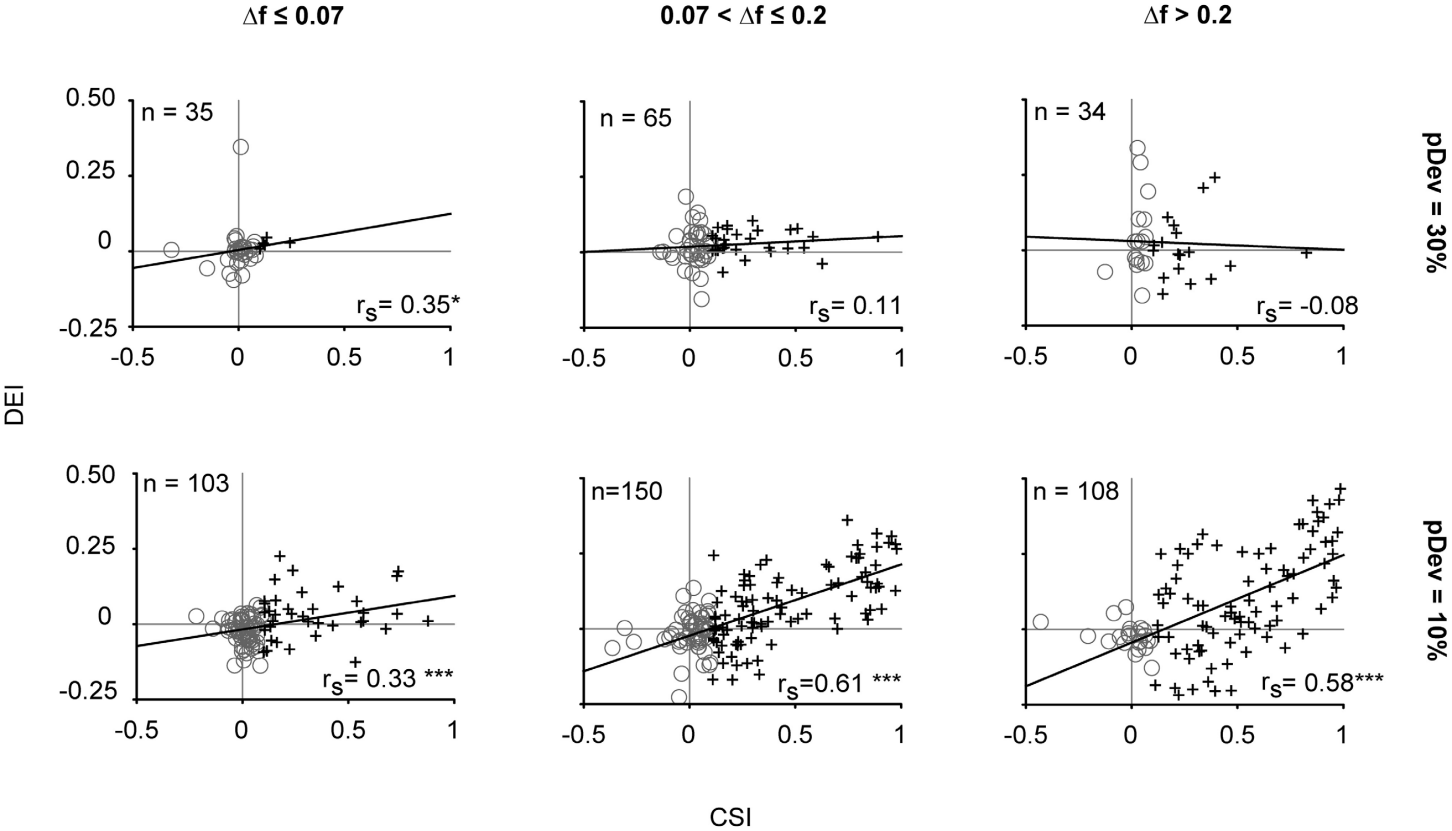
**The stimulus discriminability reflects the degree of stimulus-specific adaptation exhibited by IC neurons.** Correlation of the stimulus discriminability enhancement (DEI) and the SSA index (CSI). In gray circles are represented the neurons with CSI ≤ 0.1 and in dark crosses those with CSI > 0.1. The linear correlation was reflected by the Spearman's correlation coefficient (*r*_*s*_), whose strength varied according to the frequency contrast (columns) and probability of the deviant tone (rows). ^*^p < 0.05, ^***^p < 0.001.

Finally, we analyzed the relationship between the two metrics used to quantify the neuronal responses in order to explore whether or not the change in stimulus discrimination can be predicted by their SSA index. This analysis demonstrated a strong positive correlation between the degree of adaptation (CSI) and the enhancement in the frequency discriminability (DEI) shown by neurons under the condition with the lowest deviant probability, that is, when pDev = 10% (Spearman's rho; *p* < 0.001) (Figure 8). The great majority of neurons with CSI < 0.1 had discrimination indices clustered around the origin (gray circles). By contrast, most neurons with CSI > 0.1 (crosses) had a positive DEI, indicating that adapting neurons had better frequency discrimination for oddball sequences, and furthermore, there was a tendency for larger CSI values to be associated with larger DEI values.

### Relation between the width of frequency tuning and the SSA or discriminability exhibited by IC neurons

Previous reports demonstrated a differential expression of SSA through the lemniscal and non-lemniscal subdivisions of the IC (Pérez-González et al., 2005; Malmierca et al., 2009; Ayala and Malmierca, 2012; Duque et al., 2012) and medial geniculate body (MGB) (Antunes et al., 2010) of the rat. Neurons in the cortical regions of the IC exhibit broader FRAs than the ones from the central nucleus and the broader the response area is, the higher the SSA levels are (Duque et al., 2012). In order to test whether or not this relationship is found in our neuronal sample, we analyzed the width of response areas as a function of the level of SSA.

**Figure 9:**
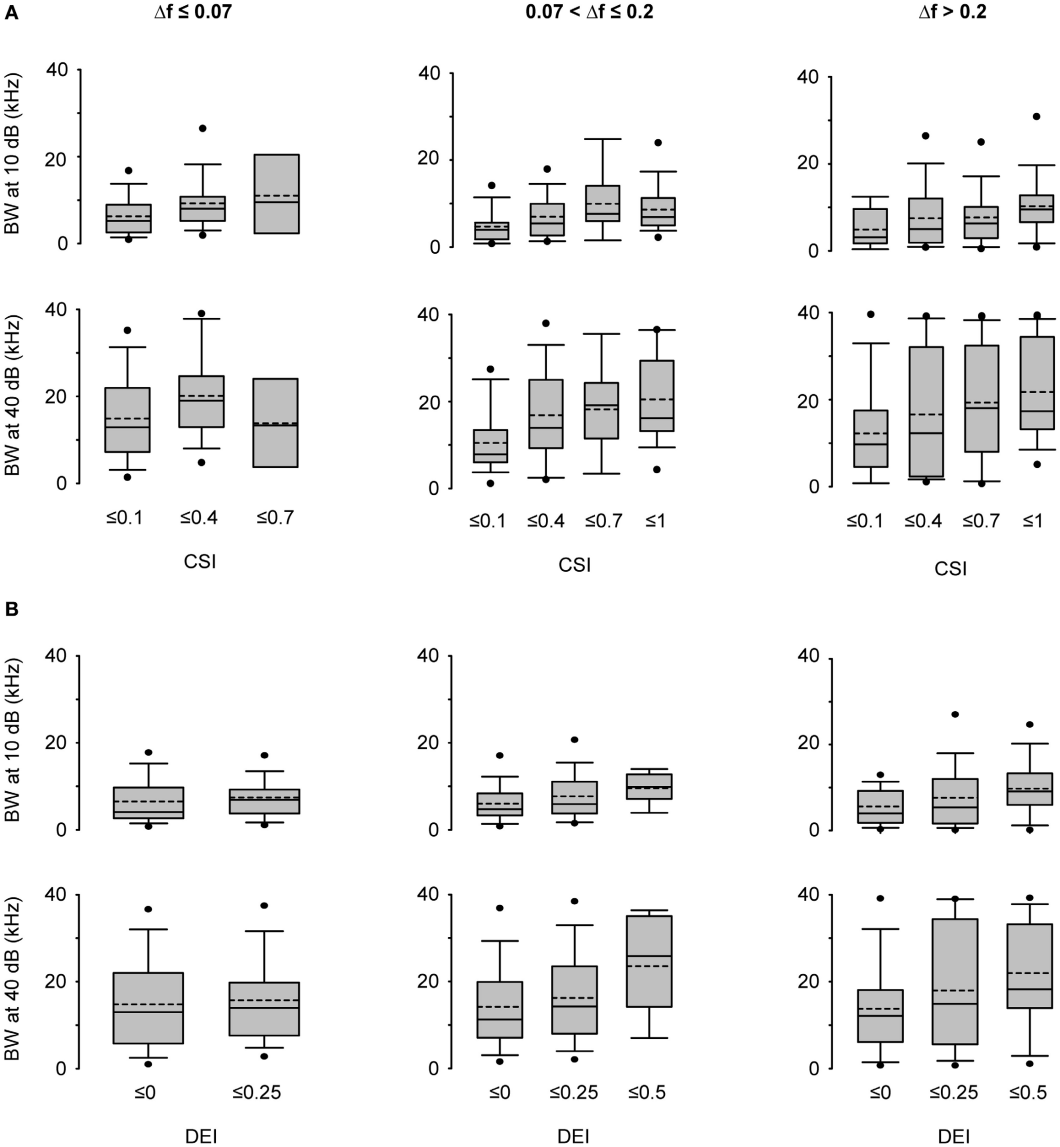
**Bandwidth of frequency response areas and SSA level of IC neurons. (A)** Box plots of the bandwidth (BW) values grouped into CSI ranges for the three Δ*f* intervals with the mean (dashed line) and median values (solid line) indicated, as well as, the 5^th^ and 95^th^ outliers. There is an increment in the bandwidths as neurons have higher CSI [*F*_(1, 688)_ = 37.5, *p* = 0] and as the level increased [*F*_(1, 688)_ = 77.4, *p* = 0] (Analysis of covariance of BW with level, Δ*f* and CSI as factors). **(B)** Box plots of BW values grouped into DEI ranges. The BW increases for neurons that displayed higher discriminability improvement under the oddball condition [*F*_(1, 688)_ = 26.7, *p* = 0]. Same format as panel **(A)**. (Analysis of covariance of BW with level, Δ*f* and DEI as factors).

Figure 9A displays the bandwidths at 10 and 40 dB SPL above threshold (BW_10, 40_, respectively) for the lowest deviant probability (pDev = 10%) as a function of the CSI. The group of CSI ≤ 0.1 included all neurons that were considered to lack SSA. The other CSI cutoffs were selected to have approximately equal-size groups. It is interesting to note that there were neurons with very broad bandwidth already at 10 dB above threshold. We performed an analysis of covariance of BW, with level above threshold (10 or 40 dB SPL) and frequency separation as qualitative factors and CSI as a continuous factor. We found a highly significant effect of CSI [*F*_(1, 688)_ = 37.5, *p* = 0]. The slope of the dependence of BW on CSI indicated that BW increased on average by 6.6 kHz as CSI increased from zero to one. The main effect of frequency separation was not significant, [*F*_(2, 688)_ = 0.54, *p* = 0.6], while the level above threshold had, as expected, a significant effect on BW [*F*_(1, 688)_ = 77.4, *p* = 0]. There was a significant interaction between the CSI slope and level above threshold [*F*_(1, 688)_ = 5.8, *p* = 0.01], and *post-hoc* comparison indicated that CSI slopes at 10 and 40 dB above threshold were significantly different (*p* < 0.05).

As expected from the positive correlation between DEI and CSI (Figure 8), a significant effect of DEI on BW was also found [*F*_(1, 688)_ = 26.7, *p* = 0] (Figure 9B). In consequence, a greater neuronal discriminability in the oddball condition is associated with a wider frequency integration range. DEI also had significant interaction with level above threshold [*F*_(1, 688)_ = 3.9, *p* = 0.048] (analysis of covariance of BW with level, frequency separation, and now with DEI as a continuous factor).

**Figure 10:**
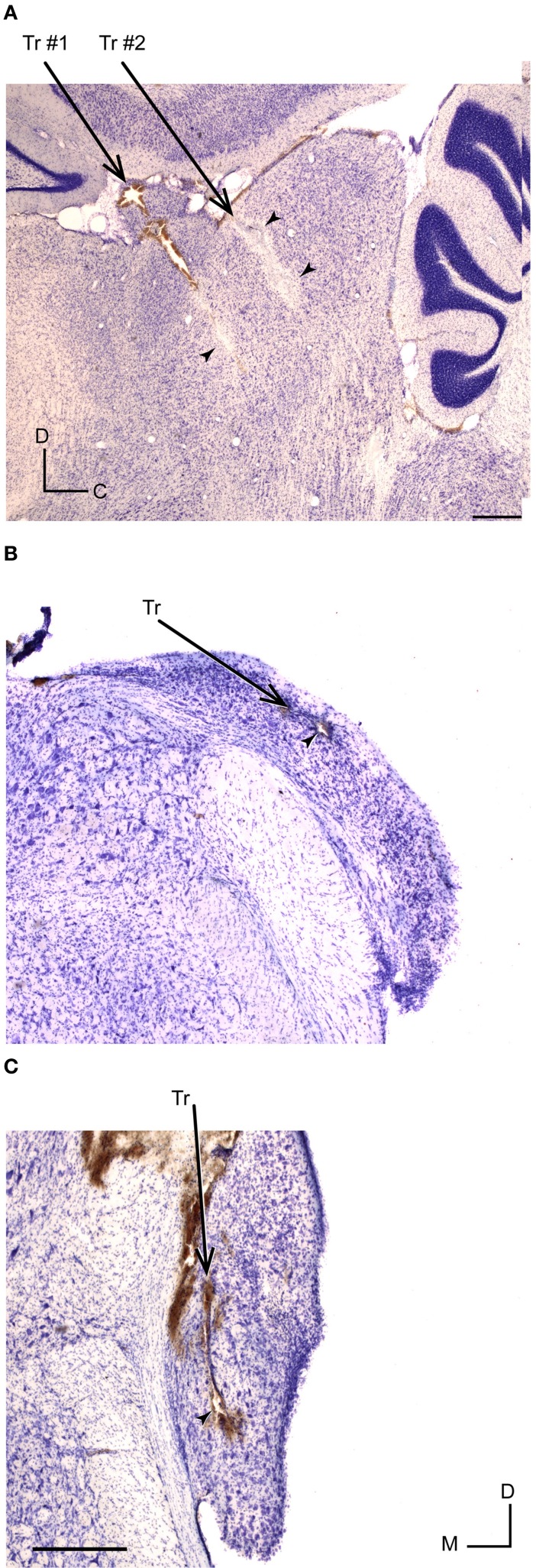
**Histological identification of recording sites of IC and CN neurons. (A)** Example of recording sites marked with an electrolytic lesion (arrowheads) in the lateral cortex of the IC at 1.9 mm lateral, according to Paxinos and Watson (2007). Two different tracts are indicated by Tr #1 and Tr #2. **(B,C)** Recording sites (arrowheads) and tracts (Tr) located in DCN (at 11.52 mm from bregma) and VCN (at 11.04 mm from bregma), respectively. The slices were Nissl stained and cut at 40 μm in a sagital **(A)** and coronal plane **(B,C)**. Scale bars of 500 μm. D, dorsal; C, caudal; M, medial.

In selected cases, we made electrolytic lesions in the IC and determined that we recorded neurons from central nucleus (*n* = 9) and from cortical regions (*n* = 16). Within this very limited sample, the central nucleus neurons had a CSI of 0.11 ± 0.21 and a DEI of −0.001 ± 0.12 (median ± SD). For the cortical neurons, the CSI and DEI were of 0.34 ± 0.3 and of 0.03 ± 0.13, respectively. However, this number of histological localizations was insufficient to guarantee a reliable study to correlate SSA and discriminability degree across the different subdivisions of the IC. Figure 10A showed an example of a typical lesion located in the lateral cortex of the IC (Loftus et al., 2008; Malmierca et al., 2011).

### CN neurons do not exhibit SSA and their frequency discriminability is not sensitive to a probability context

Since SSA is present in the IC, we wanted to explore whether SSA is already ubiquitously expressed earlier. We recorded 51 CN neurons to test whether SSA is exhibited by single-units and if so, whether adaptation strength correlates with neuronal sensitivity as shown for the IC neurons.

**Figure 11:**
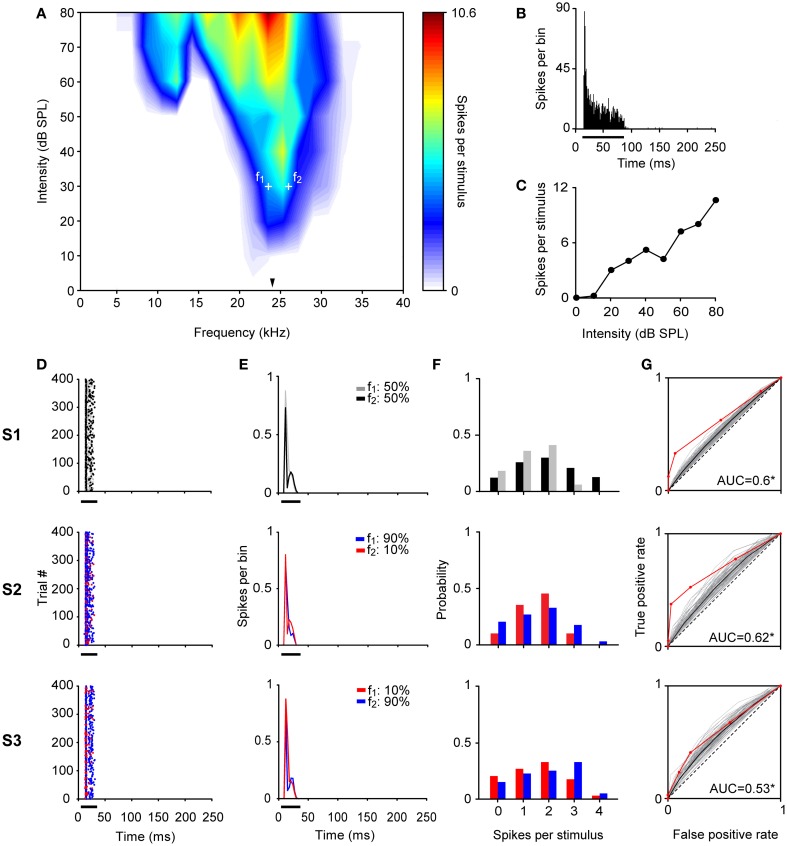
**Example of a CN neuron.** The format for all panels is the same as in Figures 3–5. **(A)** The BF was 23.4 kHz (indicated by the arrowhead) and the tested frequencies (*f*_1_ = 23.4 kHz and *f*_2_ = 25.9 kHz, white crosses) differ by 0.144 octaves. The PSTH of the accumulated response to all the frequencies (0.5 – 40 KHz) and intensities (0 – 80 dB SPL) presented (1 ms bins), as well as, the rate-level function at BF are shown in **(B)** and **(C)**, respectively. The neuron exhibited a low CSI = 0.1 (Bootstrapping; *p* > 0.05) with a robust response across trials **(D,E)** and its frequency discriminability was not sensitive to the oddball condition **(F,G)**. The AUC values in all conditions were slightly higher than 0.5 (Permutation test; ^*^p < 0.05).

A total of 44 neurons out of 51 were localized and assigned to the ventral cochlear nucleus (VCN) (*n* = 10) or DCN (*n* = 34). The histological reconstruction for the remaining 7 neurons was not possible. Figure 10B shows the electrolytic lesion in a Nissl-stained section, illustrating the recording site of the neuron displayed in Figure 11 and located in the DCN. Another example of recording site, in the VCN, is shown in Figure 10C. The recorded neurons had a wide variety of firing patterns and rate-level functions, as have been described in detail before (Stabler et al., 1996). More than the half of neurons in the DCN (21/34) displayed non-monotonic rate-level functions (5/10 in the VCN). Our sample of DCN neurons included chopper (*n* = 13), primary like (*n* = 10), pause/build (*n* = 6), and onset (*n* = 5) firing patterns. In the VCN, all firing patterns except the choppers were present (primary like, *n* = 5; pause/build, *n* = 2; onset, *n* = 3). Figure 11 shows the response of a DCN neuron with a typical V-shaped FRA with a low-frequency tail. The evoked activity was robust across the 400 trials of the equiprobable (S1) and deviant sequences (S2, S3). The neuron showed significant discriminability under the equiprobable condition (AUC(S1) = 0.6, *p* < 0.05) which did not greatly improve under the oddball sequences (AUC(S2) = 0.62, AUC(S3) = 0.53, *p* < 0.05). This neuron failed to show SSA at a repetition rate of 4 Hz, as well as at faster stimuli presentation rates of 8 and 20 Hz (CSI = 0, *p* > 0.05).

**Figure 12:**
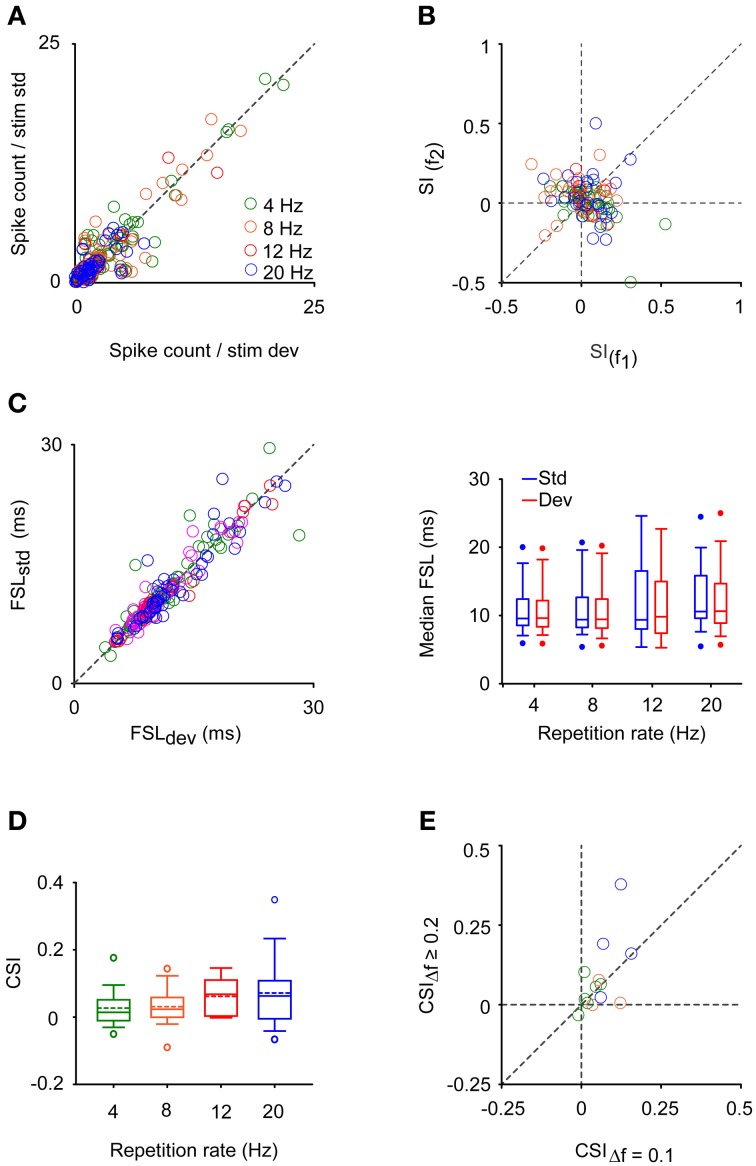
**CN neurons do not exhibit SSA. (A)** Spike count to deviant (dev) and to standard (std) stimuli elicited at different repetition rates represented in a color code. This color code is the same for **(B–E)** panels. (4 Hz, *n* = 47; 8 Hz, *n* = 29; 12 Hz, *n* = 9; 20 Hz, *n* = 26) **(B)** Scattergraph of the Frequency-Specific SSA indices (SI) for *f*_1_ and *f*_2_ presented at different repetitions rates. This index reflects the normalized spikes counts elicited when each frequency was the deviant tone regarding the response evoked when it was the standard one. **(C)** Scattergraph of the median first spike latency (FSL) for *f*_1_ and *f*_2_ when they were the deviant (FSL_dev_) or the standard (FSL_std_) stimulus. In the right column, are displayed the box plots of the median FSL for the population of neurons to deviant (red) and to standard (blue) stimulus across repetition rates. The *n* for this panel is the double of the number of neurons tested, since two frequencies were tested as deviant and as standard stimulus for each neuron. **(D,E)** Box plots of the Common**–**SSA index (CSI) when increasing the rate of stimulation and scattergraph of CSI when varying the frequency separation factor (Δ*f*), respectively.

SSA was not present in the neuronal population recorded in CN (Figure 12). We used faster repetition rates than in the IC since SSA seems to increase monotonically with stimulation rate (Malmierca et al., 2009; Antunes et al., 2010; Zhao et al., 2011; Patel et al., 2012). Regardless of the extreme repetitions rates, the strength of the neuronal response was equal for deviants and for standards stimuli (Signed Rank Test; *p* > 0.05) (Figure 12A) resulting in SI values clustered around zero (Figure 12B). There were no differences between the CSIs elicited by VCN and DCN neurons for any repetition rate tested (Rank Sum Test; *p* > 0.05). FSL is also affected by probability condition in IC, being shorter to the deviant stimulus regardless of the frequency tested (*f*_1_ or *f*_2_) (Malmierca et al., 2009; Pérez-González and Malmierca, 2012; Pérez-González et al., 2012). For CN neurons, the vast majority of FSL to deviant and to standard was almost equal and no differences in the median FSL between them was observed (Signed Rank Test; *p* > 0.05) (Figure 12C). The median of the FSLs was 9.62 ± 4.9 ms (range: 3.4 – 29.5 ms) and 9.82 ± 4.7 ms (range: 3.9 – 28.2 ms) for deviant and standard tone, respectively. These latencies are clearly shorter than the latencies of IC neurons (FSL to deviant: 26.1 ± 13.2 ms; range: 7.5 – 72 ms, FSL to standard: 29.6 ± 13.2 ms, range: 7.3 – 74.5 ms; from Malmierca et al., 2009). Although some neurons showed significant CSI > 0.1 (0.11 – 0.28) at 4 (*n* = 3), 8 (*n* = 4), 12 (*n* = 2), and 20 Hz (*n* = 5) (most of them from the DCN, *n* = 5), the average SSA indices were not significantly different from zero (Signed Rank Test; *p* > 0.05) nor they were sensitive to the rate of stimulation (Kruskal–Wallis Test; *p* > 0.05) (Figure 12D). Finally, CSI was not affected by increasing the frequency separation from Δ*f* = 0.1 to Δ*f* ≥ 0.2 (0.2 − 0.37) (Signed Rank Test; *p* > 0.05) (Figure 12E).

**Figure 13:**
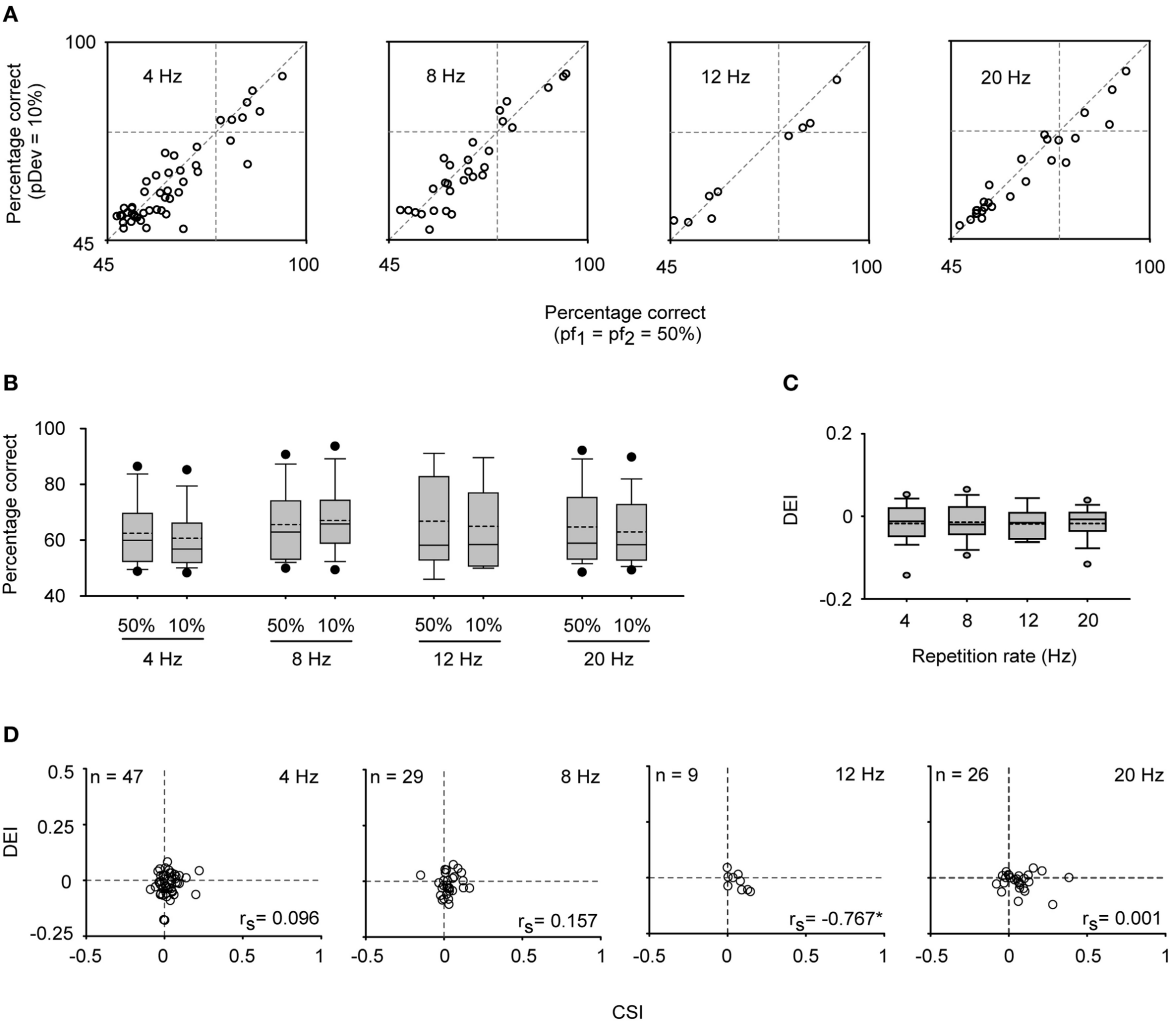
**Stimulus sensitivity in CN neurons. (A)** Neurometric boxes displaying the percentage of correct identifications in the equiprobable and oddball condition (pDev = 10%) for different repetitions rates (4, 8, 12, and 20 Hz). **(B)** Box plots indicating that no improvement was elicited under the oddball condition regarding the equiprobable one (Signed Rank Test; *p* > 0.05). **(C)** Box plots that indicate that the discrimination enhancement indexes (DEI) remained at zero and did not change across repetition rate (Kruskal–Wallis Test; *p* > 0.05). **(D)** No positive correlation between the CSI and DEI was found (Spearman's correlation coefficient, *r*_*s*_; ^*^p < 0.05).

In parallel with the lack of SSA, frequency discrimination was not affected by changes in tone probability in this neuronal population. The estimated correct detection in the oddball condition remained very similar to that elicited in the equiprobable one for most of the CN neurons (Figure 13A), and no improvement in frequency discriminability was elicited at the population level for any repetition rate group (Signed Rank Test; *p* > 0.05) (Figure 13B). Thus, the DEI was essentially zero and insensitive to increments in the repetition rate (Kruskal–Wallis Test; *p* > 0.05) (Figure 13C). As expected, it was not correlated with the SSA index (Spearman's correlation) (Figure 13D).

## Discussion

Our study demonstrates that sensitivity to frequency in IC neurons but not in CN neurons depends on probability context. Changes in frequency discriminability in IC neurons reflected the level of SSA they exhibit. Both the CSI and DEI values increased with frequency separation and DEI tended to be positive (Figures 7 and 8). The lack of effect of probability context in CN was related to the lack of SSA in the neuronal sample we recorded from (Figure 12).

### Strong SSA is exhibited by IC neurons but not by CN neurons

The strength of SSA reported here is similar to that reported previously by Malmierca et al. (2009) for IC neurons. This is not surprising, since we used a similar experimental preparation including animal model, parameters, and paradigm of stimulation (presentation rate: 4 Hz; tone duration: 75 ms; random presentation of tones). Other studies also have examined SSA in the IC, although as these studies have used different stimulation paradigms (e.g., Pérez-González et al., 2005; Lumani and Zhang, 2010), different metrics to quantify SSA (Pérez-González et al., 2005) or different stimulus repetition rates (Zhao et al., 2011), a quantitative comparison is difficult. According to the sample of histological verifications of the recording sites (Figure 10A) and taking into account the distribution of CSI (Figure 2), we recorded neurons from the central nucleus, as well as from the cortical non-lemniscal regions of IC. SSA varies as a continuum throughout the entire IC and it is strong and widespread in the non-lemniscal regions of the IC (Malmierca et al., 2009; Duque et al., 2012) and MGB (Antunes et al., 2010), being low or almost absent in the lemniscal subdivisions, the central nucleus of the IC and ventral MGB. Also, the neurons in the cortex of the IC exhibit broader response areas than those from the central nucleus (Malmierca et al., 2008, 2009; Geis et al., 2011; Duque et al., 2012). In agreement with these results, we show here that neurons with wider bandwidths (values as high as 30–40 kHz) showed the strongest SSA (Figure 9). Thus, the convergence of ascending, narrowly tuned frequency inputs with different frequency selectivity could be a major mechanism underlying SSA. In support to this idea, Taaseh et al. (2011) and Mill et al. (2011) showed that individual inputs showing simple fatigue could result in SSA. Beyond this mechanism, SSA could be further refined through the local inhibitory circuits and descending inputs from higher auditory centers. In this respect, a modulatory role of postsynaptic GABA_A_ receptors in shaping SSA in the IC has already been demonstrated (Pérez-González and Malmierca, 2012; Pérez-González et al., 2012).

Considering that (1) the IC is the locus of convergence for most inputs originating at lower auditory brainstem nuclei and the locus where the lemniscal and non-lemniscal pathways appears (Malmierca et al., 2003; Lee and Sherman, 2010, 2011), and that (2) our results demonstrated the lack of widespread SSA at the CN (Figure 12), it is tempting to suggest that cells exhibiting strong SSA in the subcortical pathways first emerge in the non-lemniscal IC. Two possible confounds currently limit this hypothesis. First, a decrease in the responsiveness and changes in the response variability of auditory cortical neurons caused by the anesthesia (Kisley and Gerstein, 1999; Harris et al., 2011) could also result in the absence of strong SSA in CN. This would be the case, for example, if SSA in the CN were dependent on descending projections for its generation. However, this possibility seems unlikely since previous studies have demonstrated that SSA at the IC (Anderson and Malmierca, 2013) and MGB (Antunes and Malmierca, 2011) persist even if the corticofugal pathway is reversibly deactivated. Second, the CN has multiple distinct physiological response types which are well-correlated with anatomical and cellular characteristics. While neurons were recorded in both VCN and DCN, currently there is no detailed classification of CN neurons in the anesthetized rat. Therefore, we cannot rule out that we recorded from all response types in this study. Indeed, because across-frequency integration seems to be important in SSA, CN neuronal types that show frequency convergence and that project to the IC, e.g., some multipolar cells or small cells from the cap area (Winter and Palmer, 1995; Jiang et al., 1996; Palmer et al., 1996; Malmierca et al., 2002; Cant and Benson, 2003) might potentially be capable of showing high levels of SSA as well. To rule out this possibility a detailed morphological and physiological study is necessary in the future. Finally, the presence of SSA in brainstem nuclei between the CN and IC also remains to be tested.

### Neuronal sensitivity of IC

We show here that the vast majority of IC neurons discriminate between nearby tones around BF even when they occur with equal probability (Figures 6A,B; upper left quadrants). While many of the AUCs were significantly larger than 0.5, they also tended to be smaller than 0.71, the standard definition of a psychophysical threshold. Note that other pairs of frequencies within the FRA with the same frequency difference could give rise to larger AUCs. Thus, our results for the equiprobable case should be seen as a lower bound on the frequency discrimination capabilities of IC neurons. Even with the biased selection of frequencies to test, a small population reached AUC > 0.71 for frequency separations as small as Δ*f* = 0.07, very close to the psychophysical thresholds of rats (e.g., Talwar and Gerstein, 1998, 1999). Interestingly, these neurons also showed narrower bandwidths than the rest. The narrow bandwidth could result in large changes in firing rates for nearby frequencies, leading to the high AUCs (Gordon et al., 2008). Such narrowly-tuned inputs could also account for the hyperacuity of MGB and cortical neurons in oddball conditions, as previously reported (Ulanovsky et al., 2003; von der Behrens et al., 2009).

Frequency discrimination in IC depended on context, being larger when the stimuli had decreased probability (Figures 6B,C and 7). Robust increases in discrimination in the oddball conditions occurred for the lower deviant probability (pDev = 10%) and the larger frequency separations (Δ*f* > 0.07). Note that we used here the mean AUC across the two oddball sequences, rather than the maximal one as used previously (Ulanovsky et al., 2003; Malmierca et al., 2009). The mean AUC is a more conservative estimate of frequency discriminability, and its use may explain why we did not observe extreme discrimination performance as reported previously in IC (Malmierca et al., 2009 their Figure 7, neurons in the upper left corner). Either way, these results emphasize the influence of context on sensory processing as early as in the IC as has been demonstrated before for the processing of interaural phase (Spitzer and Semple, 1991, 1993, 1998; McAlpine et al., 2000), level differences (Sanes et al., 1998), monaural frequency transitions (Malone and Semple, 2001) and simulated motion (Wilson and O'Neill, 1998).

### Frequency discriminability enhancement reflects the degree of SSA

We found a strong correlation between the discriminability enhancement and the degree of SSA, but only for the condition with the lowest deviant probability (pDev = 10%) and larger frequency separation (Δ*f* > 0.07) (Figure 8). These are also the conditions that had higher CSI. This positive correlation is expected from the design of the experiment. The two frequencies were selected to evoke equivalent responses in the equiprobable condition, and in the oddball condition they were expected to evoke different responses. In consequence, we expected a substantial overlap between the spike count distributions in the equiprobable condition, but a decreasing overlap in the oddball condition. Indeed, DEI depended on deviant probability and frequency separation very similarly to CSI (Figure 7). Finally, the absence of SSA and null enhancement in deviant detectability by CN neurons reinforced the notion that deviant discriminability is a functional consequence of SSA (Figures 12 and 13).

Nevertheless, we also found that neurons with CSI ≤ 0.1 showed a significant decrease in AUC in the oddball condition (Figure 6B). This decrease was due to larger corrections for the AUCs obtained under the oddball condition than to the AUCs under the equiprobable one. This trend should be seen as a negative bias in the estimation of the AUC under oddball conditions. Given that as a rule AUC increased with decreasing deviant probability, our conclusions should be considered as conservative.

### Functional significance

Neuronal responses in auditory cortex are plastic at many different time scales (Condon and Weinberger, 1991; Kilgard and Merzenich, 1998, 2002; Fritz et al., 2003; Ulanovsky et al., 2003; Froemke et al., 2007). Here we demonstrate that neurons in IC show some sort of short-term plasticity under similar conditions to neurons in MGB and A1. As previously suggested (Antunes et al., 2010), the non-lemniscal regions of the IC could transmit SSA to the non-lemniscal MGB neurons, which in turn would project to the superficial layers of AC (Cetas et al., 1999; Huang and Winer, 2000; Anderson et al., 2009). Neurons in the medial division of the MGB have large-diameter axons that are known to terminate primarily in layer I of the auditory cortex in both primary and secondary cortical fields. For example, in the somatosensory cortex Cauller and Connors (1994) observed strong excitatory effects on pyramidal cells present in layers II, III and V to be mediated by long horizontal axons located in layer I. Further experiments are required in order to check this possibility. Thus, at all levels of the auditory pathways, context-dependence of the responses could serve for adjusting the neural code to match the statistics of the input signal to produce an efficient representation of auditory scene. Similarly, the changes in responses as a function of tone probability could serve in the processes of auditory scene analysis. Indeed, auditory stream segregation is also sensitive to frequency separation and presentation rate (e.g., Fishman et al., 2004; Fishman and Steinschneider, 2010). Moreover, there is evidence suggesting the involvement of pre-attentive neural process in auditory stream segregation (Winkler et al., 2003). Thus, SSA in IC may increase the saliency of low-probability signals, helping to segregate them by reducing the ambiguity of the neuronal representations for downstream read-out mechanisms.

Interestingly, our results suggest that the initial locus for the computation of SSA is not at the very first stations of the auditory pathway, e.g., the CN. Thus, the picture of the auditory system that emerges here reinforces the idea that the initial coding of sounds is purely based on their short-term physical characteristics, and sensitivity to longer contexts that is required for higher-order processing, efficient coding, and auditory scene analysis appears only later.

## Author contributions

Manuel S. Malmierca and David Pérez-González designed research; Yaneri A. Ayala performed research; Yaneri A. Ayala, Daniel Duque, David Pérez-González and Israel Nelken analyzed data; Yaneri A. Ayala, David Pérez-González, Israel Nelken and Manuel S. Malmierca wrote the paper.

### Conflict of interest statement

The authors declare that the research was conducted in the absence of any commercial or financial relationships that could be construed as a potential conflict of interest.

## References

[B1] Anderson L. A. Christianson G. B. Linden J. F. (2009). Stimulus-specific adaptation occurs in the auditory thalamus. J. Neurosci. 29, 7359–7363 10.1523/JNEUROSCI.0793-09.2009 19494157 PMC6666468

[B2] Anderson L. A. Izquierdo M. A. Antunes F. M. Malmierca M. S. (2009). A monosynaptic pathway from dorsal cochlear nucleus to auditory cortex in rat. Neuroreport 20, 462–466 10.1097/WNR.0b013e328326f5ab 19240662

[B3] Anderson L. A. Malmierca M. S. (2013). The effect of auditory cortical deactivation on stimulus-specific adaptation in the inferior colliculus of the rat. Eur. J. Neurosci. 37, 52–62 10.1111/ejn.12018 23121128

[B4] Antunes F. M. Malmierca M. S. (2011). Effect of auditory cortex deactivation on stimulus-specific adaptation in the medial geniculate body. J. Neurosci. 31, 17306–17316 10.1523/JNEUROSCI.1915-11.2011 22114297 PMC6623836

[B5] Antunes F. M. Nelken I. Covey E. Malmierca M. S. (2010). Stimulus-specific adaptation in the auditory thalamus of the anesthetized rat. PLoS ONE 5:e14071 10.1371/journal.pone.0014071 21124913 PMC2988819

[B6] Ayala Y. A. Malmierca M. S. (2012). Stimulus-specific adaptation and deviance detection in the inferior colliculus. Front. Neural Circuits 6:89 10.3389/fncir.2012.00089 PMC3547232 23335883

[B7] Bäuerle P. von der Behrens W. Kössl M. Gaese B. H. (2011). Stimulus-specific adaptation in the gerbil primary auditory thalamus is the result of a fast frequency-specific habituation and is regulated by the corticofugal system. J. Neurosci. 31, 9708–97022 10.1523/JNEUROSCI.5814-10.2011 21715636 PMC6623171

[B8] Cant N. B. Benson C. G. (2003). Parallel auditory pathways: projection patterns of the different neuronal populations in the dorsal and ventral cochlear nuclei. Brain Res. Bull. 60, 457–474 10.1016/S0361-9230(03)00050-9 12787867

[B9] Cauller L. J. Connors B. W. (1994). Synaptic physiology of horizontal afferents to layer I in slices of rat SI neocortex. J. Neurosci. 14, 751–762 7905516 10.1523/JNEUROSCI.14-02-00751.1994 PMC6576808

[B10] Cetas J. S. de Venecia R. K. McMullen N. T. (1999). Thalamocortical afferents of Lorente de Nó: medial geniculate axons that project to primary auditory cortex have collateral branches to layer I. Brain Res. 830, 203–208 10.1016/S0006-8993(99)01355-4 10350577

[B11] Chechik G. Anderson M. J. Bar-Yosef O. Young E. D. Tishby N. Nelken I. (2006). Reduction of information redundancy in the ascending auditory pathway. Neuron 51, 359–368 10.1016/j.neuron.2006.06.030 16880130

[B12] Cohn T. E. Green D. G. Tanner W. P. Jr. (1975). Receiver operating characteristic analysis. Application to the study of quantum fluctuation effects in optic nerve of Rana pipiens. J. Gen. Physiol. 66, 583–616 172597 10.1085/jgp.66.5.583 PMC2226222

[B13] Condon C. D. Weinberger N. M. (1991). Habituation produces frequency-specific plasticity of receptive fields in the auditory cortex. Behav. Neurosci. 105, 416–430 1863363 10.1037//0735-7044.105.3.416

[B14] Duque D. Pérez-González D. Ayala A. Y. Palmer A. R. Malmierca M. S. (2012). Topographic distribution, frequency and intensity dependence of stimulus specific adaptation in the inferior colliculus of the rat. J. Neurosci. 32, 17762–17774 10.1523/JNEUROSCI.3190-12.2012 23223296 PMC6621662

[B15] Faure P. A. Fremouw T. Casseday J. H. Covey E. (2003). Temporal masking reveals properties of sound-evoked inhibition in duration-tuned neurons of the inferior colliculus. J. Neurosci. 23, 3052–3065 12684492 10.1523/JNEUROSCI.23-07-03052.2003 PMC6742117

[B16] Fawcett T. (2006). An introduction to ROC analysis. Pattern Recognit. Lett. 27, 861–874

[B17] Fishman Y. I. Arezzo J. C. Steinschneider M. (2004). Auditory stream segregation in monkey auditory cortex: effects of frequency separation, presentation rate, and tone duration. J. Acoust. Soc. Am. 116, 1656–1670 10.1121/1.1778903 15478432

[B18] Fishman Y. I. Steinschneider M. (2010). Formation of auditory streams, in The Oxford Handbook of Auditory Science: the Auditory Brain, ed Moore D. R. (New York, NY: Oxford UP), 215–245

[B19] Fritz J. Shamma S. Elhilali M. Klein D. (2003). Rapid task-related plasticity of spectrotemporal receptive fields in primary auditory cortex. Nat. Neurosci. 6, 1216–1223 10.1038/nn1141 14583754

[B20] Froemke R. C. Merzenich M. M. Schreiner C. E. (2007). A synaptic memory trace for cortical receptive field plasticity. Nature 450, 425–429 10.1038/nature06289 18004384

[B21] Geis H. R. van der Heijden M. Borst J. G. (2011). Subcortical input heterogeneity in the mouse inferior colliculus. J. Physiol. 589, 3955–3967 10.1113/jphysiol.2011.210278 21727222 PMC3179995

[B22] Gordon N. Shackleton T. M. Palmer A. R. Nelken I. (2008). Responses of neurons in the inferior colliculus to binaural disparities: insights from the use of Fisher information and mutual information. J. Neurosci. Methods 169, 391–404 10.1016/j.jneumeth.2007.11.005 18093660

[B23] Green D. M. Swets J. A. (1966). Signal Detection Theory and Psychophysics. New York, NY: Wiley

[B24] Gutfreund Y. (2012). Stimulus-specific adaptation, habituation and change detection in the gaze control system. Biol. Cybern. 106, 657–668 10.1007/s00422-012-0497-3 22711216

[B25] Huang C. L. Winer J. A. (2000). Auditory thalamocortical projections in the cat: laminar and areal patterns of input. J. Comp. Neurol. 427, 302–331 10.1002/1096-9861(20001113)427:2<302::AID-CNE10>3.0.CO;2-J 11054695

[B26] Hara K. Harris R. A. (2002). The anesthetic mechanism of urethane: the effects on neurotransmitter-gated ion channels. Anesth. Analg. 94, 313–318 11812690 10.1097/00000539-200202000-00015

[B27] Harris K. D. Bartho P. Chadderton P. Curto C. De La Rocha J. Hollender L. (2011). How do neurons work together? Lessons from auditory cortex. Hear. Res. 271, 37–53 10.1016/j.heares.2010.06.006 20603208 PMC2992581

[B28] Hernández O. Espinosa N. Pérez-González D. Malmierca M. S. (2005). The inferior colliculus of the rat: a quantitative analysis of monaural frequency response areas. Neuroscience 132, 203–217 10.1016/j.neuroscience.2005.01.001 15780479

[B29] Jiang D. Palmer A. R. Winter I. M. (1996). Frequency extent of two-tone facilitation in onset units in the ventral cochlear nucleus. J. Neurophysiol. 75, 380–395 8822565 10.1152/jn.1996.75.1.380

[B30] Kilgard M. P. Merzenich M. M. (1998). Plasticity of temporal information processing in the primary auditory cortex. Nat. Neurosci. 1, 727–731 10.1038/3729 10196590 PMC2948964

[B31] Kilgard M. P. Merzenich M. M. (2002). Order-sensitive plasticity in adult primary auditory cortex. Proc. Natl. Acad. Sci. U.S.A. 99, 3205–3209 10.1073/pnas.261705198 11880653 PMC122497

[B32] Kisley M. A. Gerstein G. L. (1999). Trial-to-trial variability and state-dependent modulation of auditory-evoked responses in cortex. J. Neurosci. 19, 10451–10460 10575042 10.1523/JNEUROSCI.19-23-10451.1999 PMC6782416

[B33] LeBeau F. E. Malmierca M. S. Rees A. (2001). Iontophoresis *in vivo* demonstrates a key role for GABA(A) and glycinergic inhibition in shaping frequency response areas in the inferior colliculus of guinea pig. J. Neurosci. 21, 7303–7312 11549740 10.1523/JNEUROSCI.21-18-07303.2001 PMC6762982

[B34] Lee C. C. Sherman S. M. (2010). Topography and physiology of ascending streams in the auditory tectothalamic pathway. Proc. Natl. Acad. Sci. U.S.A. 107, 372–377 10.1073/pnas.0907873107 20018757 PMC2806784

[B35] Lee C. C. Sherman S. M. (2011). On the classification of pathways in the auditory midbrain, thalamus, and cortex. Hear. Res. 276, 79–87 10.1016/j.heares.2010.12.012 21184817 PMC3108009

[B36] Loftus W. C. Malmierca M. S. Bishop D. C. Oliver D. L. (2008). The cytoarchitecture of the inferior colliculus revisited: a common organization of the lateral cortex in rat and cat. Neuroscience 154, 196–205 10.1016/j.neuroscience.2008.01.019 18313229 PMC2562950

[B37] Lumani A. Zhang H. (2010). Responses of neurons in the rat's dorsal cortex of the inferior colliculus to monaural tone bursts. Brain Res. 1351, 115–129 10.1016/j.brainres.2010.06.066 20615398

[B38] Malmierca M. S. Blackstad T. W. Osen K. K. (2011). Computer-assisted 3-D reconstructions of Golgi-impregnated neurons in the cortical regions of the inferior colliculus of rat. Hear. Res. 274, 13–26 10.1016/j.heares.2010.06.011 20600744

[B39] Malmierca M. S. Cristaudo S. Pérez-González D. Covey E. (2009). Stimulus-specific adaptation in the inferior colliculus of the anesthetized rat. J. Neurosci. 29, 5483–5493 10.1523/JNEUROSCI.4153-08.2009 19403816 PMC2715893

[B40] Malmierca M. S. Hernández O. Falconi A. Lopez-Poveda E. A. Merchan M. Rees A. (2003). The commissure of the inferior colliculus shapes frequency response areas in rat: an *in vivo* study using reversible blockade with microinjection of kynurenic acid. Exp. Brain Res. 153, 522–529 10.1007/s00221-003-1615-1 14508633

[B41] Malmierca M. S. Izquierdo M. A. Cristaudo S. Hernández O. Pérez-González D. Covey E. (2008). A discontinuous tonotopic organization in the inferior colliculus of the rat. J. Neurosci. 28, 4767–4776 10.1523/JNEUROSCI.0238-08.2008 18448653 PMC2440588

[B42] Malmierca M. S. Merchán M. A. Henkel C. K. Oliver D. L. (2002). Direct projections from cochlear nuclear complex to auditory thalamus in the rat. J. Neurosci. 22, 10891–10897 12486183 10.1523/JNEUROSCI.22-24-10891.2002 PMC6758437

[B43] Malmierca M. S. Ryugo D. K. (2011). Descending connections of auditory cortex to the midbrain and brainstem, in The Auditory Cortex, eds Winer J. A. Schreiner C. E. (New York, NY: Springer), 189–208

[B44] Malone B. J. Semple M. N. (2001). Effects of auditory stimulus context on the representation of frequency in the gerbil inferior colliculus. J. Neurophysiol. 86, 1113–1130 11535662 10.1152/jn.2001.86.3.1113

[B45] Maris E. (2012). Statistical testing in electrophysiological studies. Psychophysiology 49, 549–565 10.1111/j.1469-8986.2011.01320.x 22176204

[B46] McAlpine D. Jiang D. Shackleton T. M. Palmer A. R. (2000). Responses of neurons in the inferior colliculus to dynamic interaural phase cues: evidence for a mechanism of binaural adaptation. J. Neurophysiol. 83, 1356–1365 10712463 10.1152/jn.2000.83.3.1356

[B47] Merrill E. G. Ainsworth A. (1972). Glass-coated platinum-plated tungsten microelectrodes. Med. Biol. Eng. 10, 662–672 5076431 10.1007/BF02476084

[B48] Mill R. Coath M. Wennekers T. Denham S. L. (2011). A neurocomputational model of stimulus-specific adaptation to oddball and Markov sequences. PLoS Comput. Biol. 7:e1002117 10.1371/journal.pcbi.1002117 21876661 PMC3158038

[B49] Näätänen R. (1992). Attention and Brain Function. Hillsdale, NJ: Lawrence Erlbaum

[B50] Nelken I. Ulanovsky N. (2007). Mismatch negativity and stimulus-specific adaptation in animal models. J. Psychophysiol. 21, 214–223

[B51] Nelson D. A. Kiester T. E. (1978). Frequency discrimination in the chinchilla. J. Acoust. Soc. Am. 64, 114–126 711990 10.1121/1.381977

[B52] Netser S. Zahar Y. Gutfreund Y. (2011). Stimulus-specific adaptation: can it be a neural correlate of behavioral habituation? J. Neurosci. 31, 17811–17820 10.1523/JNEUROSCI.4790-11.2011 22159097 PMC6634140

[B53] Palmer A. R. Jiang D. Marshall D. H. (1996). Responses of ventral cochlear nucleus onset and chopper units as a function of signal bandwidth. J. Neurophysiol. 75, 780–794 8714652 10.1152/jn.1996.75.2.780

[B54] Patel C. R. Redhead C. Cervi A. L. Zhang H. (2012). Neural sensitivity to novel sounds in the rat's dorsal cortex of the inferior colliculus as revealed by evoked local field potentials. Hear. Res. 286, 41–54 10.1016/j.heares.2012.02.007 22406035

[B55] Paxinos G. Watson C. (2007). The Rat Brain in Stereotaxic Coordinates. Burlington, VT: Elsevier-Academic

[B56] Pérez-González D. Hernández O. Covey E. Malmierca M. S. (2012). GABA(A)-mediated inhibition modulates stimulus-specific adaptation in the inferior colliculus. PLoS ONE 7:e34297 10.1371/journal.pone.0034297 22479591 PMC3315508

[B57a] Pérez-González D. Malmierca M. S. (2012). Variability of the time course of stimulus-specific adaptation in the inferior colliculus. Front. Neural Circuits 6:107 10.3389/fncir.2012.00107 PMC3530767 23293586

[B57] Pérez-González D. Malmierca M. S. Covey E. (2005). Novelty detector neurons in the mammalian auditory midbrain. Eur. J. Neurosci. 22, 2879–2885 10.1111/j.1460-9568.2005.04472.x 16324123

[B58] Pérez-González D. Malmierca M. S. Moore J. M. Hernández O. Covey E. (2006). Duration selective neurons in the inferior colliculus of the rat: topographic distribution and relation of duration sensitivity to other response properties. J. Neurophysiol. 95, 823–836 10.1152/jn.00741.2005 16192332

[B59] Reches A. Gutfreund Y. (2008). Stimulus-specific adaptations in the gaze control system of the barn owl. J. Neurosci. 28, 1523–1533 10.1523/JNEUROSCI.3785-07.2008 18256273 PMC6671572

[B60] Reches A. Gutfreund Y. (2009). Auditory and multisensory responses in the tectofugal pathway of the barn owl. J. Neurosci. 29, 9602–9613 10.1523/JNEUROSCI.6117-08.2009 19641123 PMC6666540

[B61] Reches A. Netser S. Gutfreund Y. (2010). Interactions between stimulus-specific adaptation and visual auditory integration in the forebrain of the barn owl. J. Neurosci. 30, 6991–6998 10.1523/JNEUROSCI.5723-09.2010 20484641 PMC6632650

[B62] Rees A. (1990). A close-field sound system for auditory neurophysiology. J. Physiol. 430, 2

[B63] Rees A. Sarbaz A. Malmierca M. S. Le Beau F. E. (1997). Regularity of firing of neurons in the inferior colliculus. J. Neurophysiol. 77, 2945–2965 9212248 10.1152/jn.1997.77.6.2945

[B64] Sanes D. H. Malone B. J. Semple M. N. (1998). Role of synaptic inhibition in processing of dynamic binaural level stimuli. J. Neurosci. 18, 794–803 9425020 10.1523/JNEUROSCI.18-02-00794.1998 PMC6792525

[B65] Shackleton T. M. Arnott R. H. Palmer A. R. (2005). Sensitivity to interaural correlation of single neurons in the inferior colliculus of guinea pigs. J. Assoc. Res. Otolaryngol. 6, 244–259 10.1007/s10162-005-0005-8 16080025 PMC2504597

[B66] Shackleton T. M. Skottun B. C. Arnott R. H. Palmer A. R. (2003). Interaural time difference discrimination thresholds for single neurons in the inferior colliculus of guinea pigs. J. Neurosci. 23, 716–724 12533632 10.1523/JNEUROSCI.23-02-00716.2003 PMC6741888

[B67] Shofner W. P. (2000). Comparison of frequency discrimination thresholds for complex and single tones in chinchillas. Hear. Res. 149, 106–114 10.1016/S0378-5955(00)00171-4 11033250

[B68] Shofner W. P. Dye R. H. Jr. (1989). Statistical and receiver operating characteristic analysis of empirical spike-count distributions: quantifying the ability of cochlear nucleus units to signal intensity changes. J. Acoust. Soc. Am. 86, 2172–2184 10.1121/1.398478 2600308

[B69] Sinnott J. M. Petersen M. R. Hopp S. L. (1985). Frequency and intensity discrimination in humans and monkeys. J. Acoust. Soc. Am. 78, 1977–1985 10.1121/1.392654 4078174

[B70] Skottun B. C. Shackleton T. M. Arnott R. H. Palmer A. R. (2001). The ability of inferior colliculus neurons to signal differences in interaural delay. Proc. Natl. Acad. Sci. U.S.A. 98, 14050–14054 10.1073/pnas.241513998 11707595 PMC61165

[B71] Spitzer M. W. Semple M. N. (1991). Interaural phase coding in auditory midbrain: influence of dynamic stimulus features. Science 254, 721–724 10.1126/science.1948053 1948053

[B72] Spitzer M. W. Semple M. N. (1993). Responses of inferior colliculus neurons to time-varying interaural phase disparity: effects of shifting the locus of virtual motion. J. Neurophysiol. 69, 1245–1263 8492161 10.1152/jn.1993.69.4.1245

[B73] Spitzer M. W. Semple M. N. (1998). Transformation of binaural response properties in the ascending auditory pathway: influence of time-varying interaural phase disparity. J. Neurophysiol. 80, 3062–3076 9862906 10.1152/jn.1998.80.6.3062

[B74] Stabler S. E. Palmer A. R. Winter I. M. (1996). Temporal and mean rate discharge patterns of single units in the dorsal cochlear nucleus of the anesthetized guinea pig. J. Neurophysiol. 76, 1667–1688 8890284 10.1152/jn.1996.76.3.1667

[B76] Stüttgen M. C. Schwarz C. Jakel F. (2011). Mapping spikes to sensations. Front. Neurosci. 5:125 10.3389/fnins.2011.00125 PMC3212738 22084627

[B77] Syka J. Rybalko N. Brozek G. Jilek M. (1996). Auditory frequency and intensity discrimination in pigmented rats. Hear. Res. 100, 107–113 8922984 10.1016/0378-5955(96)00101-3

[B78] Taaseh N. Yaron A. Nelken I. (2011). Stimulus-specific adaptation and deviance detection in the rat auditory cortex. PLoS ONE 6:e23369 10.1371/journal.pone.0023369 21853120 PMC3154435

[B79] Talwar S. K. Gerstein G. L. (1998). Auditory frequency discrimination in the white rat. Hear. Res. 126, 135–150 10.1016/S0378-5955(98)00162-2 9872142

[B80] Talwar S. K. Gerstein G. L. (1999). A signal detection analysis of auditory-frequency discrimination in the rat. J. Acoust. Soc. Am. 105, 1784–1800 10.1121/1.426716 10089602

[B81] Tanner W. P. Jr. Swets J. A. (1954). A decision-making theory of visual detection. Psychol. Rev. 61, 401–409 13215690 10.1037/h0058700

[B82] Ulanovsky N. Las L. Farkas D. Nelken I. (2004). Multiple time scales of adaptation in auditory cortex neurons. J. Neurosci. 24, 10440–10453 10.1523/JNEUROSCI.1905-04.2004 15548659 PMC6730303

[B83] Ulanovsky N. Las L. Nelken I. (2003). Processing of low-probability sounds by cortical neurons. Nat. Neurosci. 6, 391–398 10.1038/nn1032 12652303

[B84] von der Behrens W. Bäuerle P. Kossl M. Gaese B. H. (2009). Correlating stimulus-specific adaptation of cortical neurons and local field potentials in the awake rat. J. Neurosci. 29, 13837–13849 10.1523/JNEUROSCI.3475-09.2009 19889995 PMC6666711

[B85] Walker K. M. Schnupp J. W. Hart-Schnupp S. M. King A. J. Bizley J. K. (2009). Pitch discrimination by ferrets for simple and complex sounds. J. Acoust. Soc. Am. 126, 1321–1335 10.1121/1.3179676 19739746 PMC2784999

[B86] Wilson W. W. O'Neill W. E. (1998). Auditory motion induces directionally dependent receptive field shifts in inferior colliculus neurons. J. Neurophysiol. 79, 2040–2062 9535967 10.1152/jn.1998.79.4.2040

[B87] Winkler I. Sussman E. Tervaniemi M. Horvath J. Ritter W. Naatanen R. (2003). Preattentive auditory context effects. Cogn. Affect. Behav. Neurosci. 3, 57–77 12822599 10.3758/cabn.3.1.57

[B88] Winter I. M. Palmer A. R. (1995). Level dependence of cochlear nucleus onset unit responses and facilitation by second tones or broadband noise. J. Neurophysiol. 73, 141–159 7714560 10.1152/jn.1995.73.1.141

[B89] Witte R. S. Kipke D. R. (2005). Enhanced contrast sensitivity in auditory cortex as cats learn to discriminate sound frequencies. Brain Res. Cogn. Brain Res. 23, 171–184 10.1016/j.cogbrainres.2004.10.018 15820626

[B90] Yu X. J. Xu X. X. He S. He J. (2009). Change detection by thalamic reticular neurons. Nat. Neurosci. 12, 1165–1170 10.1038/nn.2373 19684591

[B91] Zhao L. Liu Y. Shen L. Feng L. Hong B. (2011). Stimulus-specific adaptation and its dynamics in the inferior colliculus of rat. Neuroscience 181, 163–174 10.1016/j.neuroscience.2011.01.060 21284952

